# m5C-methylated lncRNA NR_033928 promotes gastric cancer proliferation by stabilizing GLS mRNA to promote glutamine metabolism reprogramming

**DOI:** 10.1038/s41419-023-06049-8

**Published:** 2023-08-15

**Authors:** Lang Fang, Hongxin Huang, Jialun Lv, Zetian Chen, Chen Lu, Tianlu Jiang, Penghui Xu, Ying Li, Sen Wang, Bowen Li, Zheng Li, Weizhi Wang, Zekuan Xu

**Affiliations:** 1 Department of General Surgery, The First Affiliated Hospital of Nanjing Medical University, Nanjing, Jiangsu Province China; 2 Jiangsu Key Lab of Cancer Biomarkers, Prevention and Treatment, Collaborative Innovation Center for Cancer Personalized Medicine, Nanjing Medical University, 210029 Nanjing, Jiangsu Province China

**Keywords:** Gastric cancer, Cancer metabolism

## Abstract

Abnormal 5-methylcytosine (m5C) methylation has been proved to be closely related to gastric carcinogenesis, progression, and prognosis. Dysregulated long noncoding RNAs (lncRNAs) participate in a variety of biological processes in cancer. However, to date, m5C-methylated lncRNAs are rarely researched in gastric cancer (GC). Here, we found that RNA cytosine-C(5)-methyltransferase (NSUN2) was upregulated in GC and high NSUN2 expression was associated with poor prognosis. NR_033928 was identified as an NSUN2-methylated and upregulated lncRNA in GC. Functionally, NR_033928 upregulated the expression of glutaminase (GLS) by interacting with IGF2BP3/HUR complex to promote GLS mRNA stability. Increased glutamine metabolite, α-KG, upregulated NR_033928 expression by enhancing its promoter 5-hydroxymethylcytosine (hm5C) demethylation. In conclusion, our results revealed that NSUN2-methylated NR_033928 promoted GC progression and might be a potential prognostic and therapeutic target for GC.

## Introduction

Gastric cancer (GC) is one of the most common malignant tumors, with the fourth lethality and fifth incidence in the world [1]. Almost half of the patients occurred in East Asian countries, especially in China [2]. Quite a number of patients were diagnosed with advantaged GC and had a poor diagnosis due to a deficiency of early diagnosis and screening of GC [3]. Therefore, it is essential and urgent to investigate the underlying molecular mechanisms of gastric carcinogenesis and progression.

The factors that have been proved to cause GC are infecting *Helicobacter pylori*, intaking excessive food rich in nitrite compounds, having precancerous lesions, and so on [4]. Many molecular mechanisms are identified to contribute to tumor formation and progression, which include the increase of tumor mutation burden, immune escape, epigenetic modification, and so on. Methylation modifications are one of the most common epigenetic modifications, which exist in DNA, RNA, and protein modification [5]. Our previous studies showed that METTL3-mediated m6A modification of MRP1 regulated the chemoresistance of gastrointestinal stromal tumors [6]. In addition to m6A methylation, m5C methylation has been proved to be one of the most important nucleic acid modifications and is widely expressed in non-coding RNA and mRNA with high abundance [7, 8]. Recent studies revealed that m5C methylation played important roles in cancer progression and was associated with poor prognosis [9, 10]. However, whether m5C methylation plays a role in gastric cancer needs further investigation.

LncRNAs consist of a group of long non-coding RNA with more than 200 nucleotides [11]. LncRNAs compose 4% to 9% of transcripts in mammals which is far more than the proportion of protein-coding RNAs [12]. LncRNAs were initially thought to be a “noise” from genomic transcription, a byproduct not having a biological function. However, mounting evidence showed that lncRNAs participated in a variety of physiological and pathological processes in normal tissues and cancer, which included chromatin modification, transcriptional activation, transcriptional interference, intranuclear transport, and so on [13, 14]. Our previous studies identified several oncogenic lncRNAs in GC. For example, Fan et al. reported that lncRNA CCDC144NL-AS1 acted as a competing endogenous RNA for sponging miR-143-3p and upregulated the expression MAP3K7 in GC progression [15]. However, whether lncRNAs modified by m5C methylation play a part in GC remains unknown.

There is growing evidence indicating that metabolic reprogramming plays a vital role in tumorigenesis [16]. Glutamine is one of the most abundant and important amino acids in the human body, which is especially important for rapid tumor proliferation [17]. Glutamine is first converted to glutamate by glutaminase (GLS), then to α-ketoglutarate (α-KG). α-KG can be used as a substrate of the tricarboxylic acid cycle and s nutrient for the synthesis of lipids, nucleotides, and amino acids [18]. GLS is overexpressed and exerts oncogenic effects in many types of tumors. Knockdown or inhibition of GLS significantly suppresses the proliferation of cancer cells through siRNA or small molecules [19]. Our previous work also demonstrated that microRNA-133a-3p overexpression blocked the activation of autophagy to ruin the abnormal GLS-mediated glutaminolysis and further inhibited the growth and metastasis of GC cells [20].

In this study, we found that NSUN2 was upregulated in GC and positively related to poor prognosis. Then, we identified an NSUN2-methylated lncRNA named NR_033928 with high expression in GC cells and tissues. Functional assays proved that NR_033928 promoted GC proliferation and suppressed apoptosis in vitro and in vivo. Next-generation sequencing found that NR_033928 promoted GC progression through GLS-mediated glutamine metabolism. Mechanistically, NR_033928 acted as a scaffold of the IGF2BP3/HUR complex to improve GLS mRNA stability, thereby increasing its expression. Accumulation of glutamine metabolite, α-KG promoted NR_033928 promoter hm5C demethylation to upregulate NR_033928 expression, thereby forming a positive feedback loop. Our results demonstrated that NSUN2-methylated NR_033928 played a critical role and might serve as a promising treatment target in GC.

## Results

### Elevated m5C regulator NSUN2 correlates with poor prognosis in GC

**Fig. 1:**
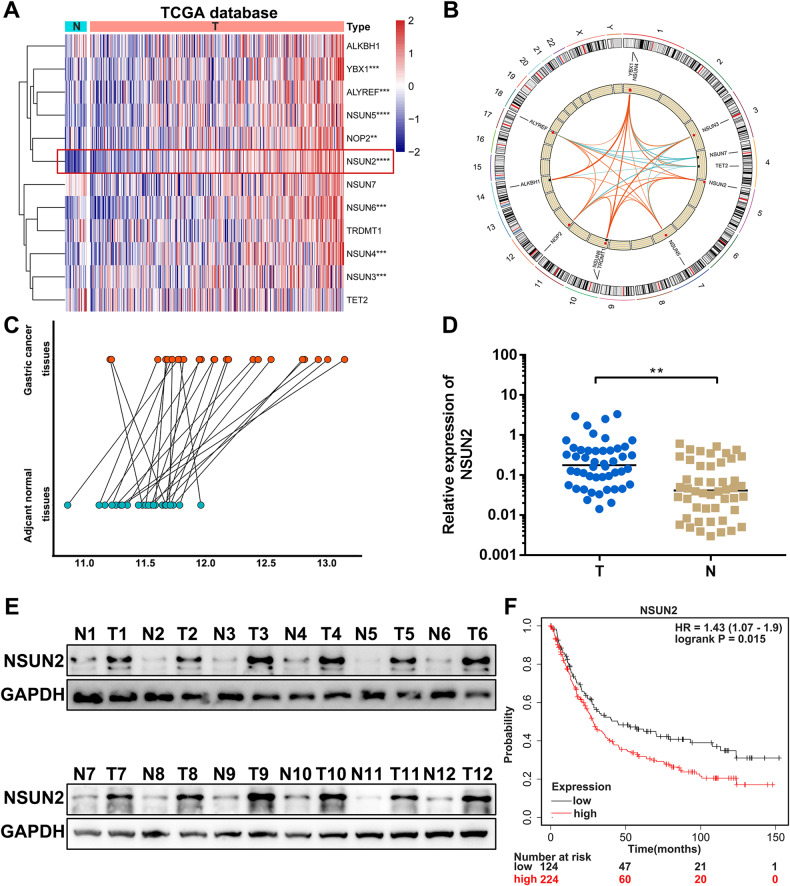
NSUN2 is upregulated and associated with poor prognosis in GC. **A** A heatmap of m5C regulators in STAD-TCGA database. **B** Chromosome position and Pearson correlation analysis of m5C regulators. **C** Relative expression of NSUN2 in 24 pairs of tumors and adjacent normal tissues microarray. **D** Relative expression of NSUN2 in 48 pairs of tumors and adjacent normal tissues. **E** NSUN2 protein levels in GC tissues and paired normal tissues. **F** Kaplan–Meier survival curves of OS based on NSUN2 expression using Kaplan–Meier Plotter. (Graph represents mean ± SD; **p < 0.01, ***p < 0.001 and ****p < 0.0001).

To explore the functional roles of m5C modification in GC, we first examined the m5C regulators expression in Stomach Adenocarcinoma (STAD) project from The Cancer Genome Atlas (TCGA) database. Results showed that m5C writers (NOP2, NSUN2, NSUN3, NSUN4, NSUN5 and NSUN6) and readers (ALYREF and YBX1) were upregulated in GC samples compared with normal tissues. And the core m5C methyltransferase NSUN2 was the most highly expressed in GC (Fig. 1A). Pearson correlation analysis indicated that NSUN2 expression was positively associated with most of the other m5C regulators’ expression (Fig. 1B). Thus, we hypothesized that NSUN2 might play oncogenic roles in GC. To verify this finding, we first analyzed NSUN2 expressing in our 24 paired GC and adjacent normal tissues microarray results. Results showed that NSUN2 was remarkably increased in GC (Fig. 1C). Then qPCR analysis performed in 48 pairs of GC and matched normal gastric tissues also validated that NSUN2 expression was significantly higher in GC (Fig. 1D). Western blot analysis showed that NSUN2 protein levels were obviously higher in GC tissues (Fig. 1E). Results from Kaplan–Meier Plotter (http://kmplot.com//analysis/) showed that NSUN2 expression was positively related to the poor overall survival of GC patients (Fig. 1F).

Altogether, these results indicated that NSUN2 was upregulated in GC and associated with poor diagnosis in GC patients.

### NR_033928 is upregulated and identified as an NSUN2-methylated lncRNA in GC

As a key cytosine-C(5)-methyltransferase, NSUN2 regulated tumorigenesis and progression by modulating the m5C modification in various RNAs, such as mRNAs and lncRNAs [21]. There have been reported that NSUN2 exerted its functions by regulating lncRNA m5C methylation in esophageal squamous cell carcinoma, hepatocellular carcinoma and cholangiocarcinoma [22–24]. However, the roles of NSUN2 methylated lncRNAs in GC are rarely clarified, which needs further investigation.

**Fig. 2:**
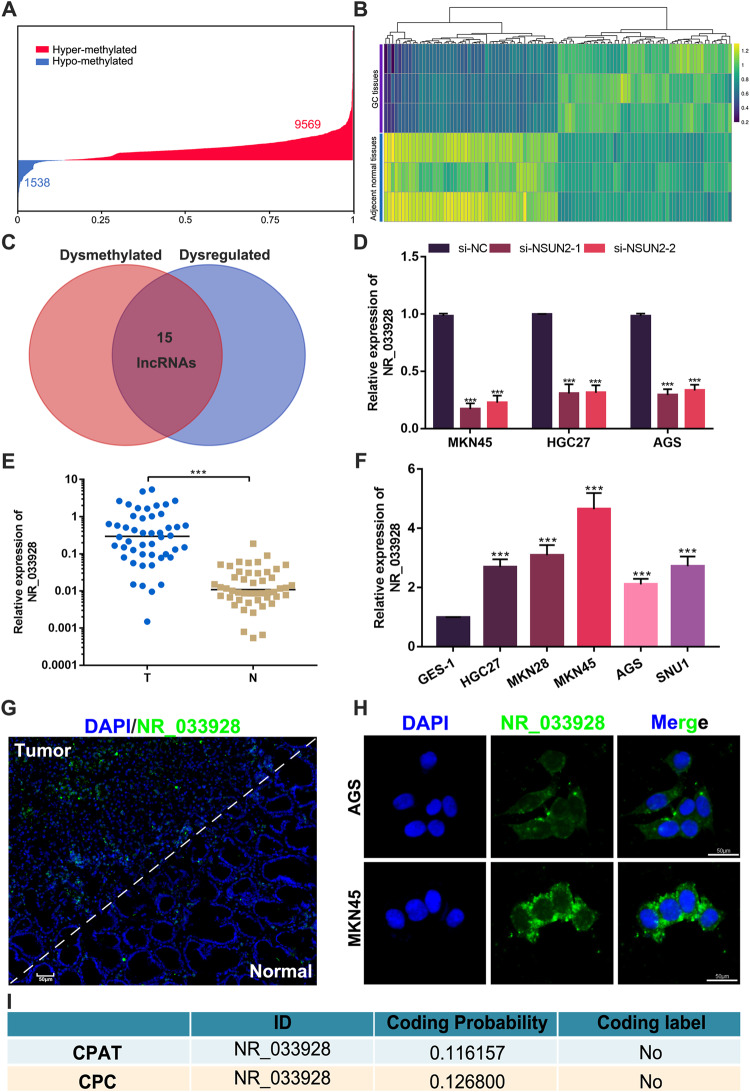
Identification and characterization of NR_033928. **A** Distribution of the different methylation levels of lncRNAs in GC tumors. **B** A heatmap showing the lncRNAs differentially expressed in three pairs of gastric cancer and adjacent normal tissues. **C** A Venn diagram showing the intersected dysmethylated and dysregulated lncRNAs in GC. **D** Relative expression of NR_033928 in GC cells transfected with NSUN2 siRNAs. **E** Relative expression of NR_033928 in 48 pairs of tumors and adjacent normal tissues. **F** Relative expression of NR_033928 in GES-1 and GC cell lines. **G** Representative FlSH image of NR_033928 in GC tissues. Scale bar = 50 μm. **H** Representative FlSH image of of NR_033928 in GC cells. Scale bar = 50 μm. **I** Predicated coding potential of NR_033928 in CPAT and CPC. (Graph represents mean ± SD; ***p < 0.001).

To map the m5C epigenetic modification of lncRNAs in GC, RNA methylation sequencing based on m5C antibodies and next-generation sequencing were conducted in three pairs of GC tissues and their adjacent normal tissues. Results showed that there were 254 dysregulated (|log2(fold change (FC)) | >1, *p* < 0.05) and 11107 differentially methylated lncRNAs(score>8, *p* < 0.001) (Fig. 2A, B). LncRNAs whose lengths were shorter than 200 bp were excluded. Further analysis showed that there were 10 upregulated and hypermethylated lncRNAs, 1 upregulated and hypomethylated lncRNA, 3 downregulated and hypermethylated lncRNAs, and 1 downregulated and hypomethylated lncRNA (Fig. 2C). To explore the potential lncRNAs modified by NSUN2, qPCR assays were performed in GC cells transfected with NSUN2 siRNAs. Results showed that NR_033928 expression was the most obviously downregulated among all candidate lncRNAs in NSUN2-silenced cells (Fig. 2D and Supplementary Fig. S1A–C). qRT-PCR was applied to confirm that the expression of NR_033928 was higher in GC tissues than matched normal tissues of 48 GC patients (Fig. 2E). RNA fluorescence in situ hybridization (RNA-FISH) assays indicated that GC tissues had a significant abundance of NR_033928 compared to adjacent normal mucosal tissues (Fig. 2G). By analyzing the patients’ clinicopathological features, we also found that the NR_033928 expression level was positively correlated with GC tumor size and TNM stage (Table 1). Then, we detected the NR_033928 expression in GC cell lines. Results showed that compared to normal human gastric cell line GES-1, the expression of NR_033928 was higher in all examined GC cell lines (HGC-27, MKN28, MKN45, AGS, and SNU1) (Fig. 2F). According to the upregulated expression level, we chose MKN45 and AGS for further study. FISH displayed that NR_033928 was mainly localized in the cytoplasm of AGS and MKN45 (Fig. 2H). CPC2 (http://cpc2.gao-lab.org/) and CPAT (http://rna-cpat.sourceforge.net/) predicted that the coding probability of NR_033928 is extremely low (Fig. 2I).

**Table 1:** Expression of NR_033928 in human gastric cancer according to patients’ clinicopathological characteristics.

Characteristics	Number	Number of patients		*p* value
		NR_033928(low)	NR_033928 (high)	
Age (years)				0.2416
<60	28	12	16	
>60	20	12	8	
Gender				0.1313
Female	17	11	6	
Male	31	13	18	
Tumor size (cm)				0.0022**
<3	16	13	3	
≥3	32	11	21	
Location				0.5509
Cardia	18	8	10	
Non-cardia	30	16	14	
T classification				0.0822
T1–T2	22	8	14	
T3–T4	26	16	10	
N classification				0.7313
N0	11	5	6	
N1–N3	37	19	18	
Clinical stage				0.0346*
I–II	17	12	5	
III–IV	31	12	19	

*p < 0.05, **p < 0.01.

Taken together, NR_033928 was identified as a potential oncogenic NSUN2-modified lncRNA in GC.

### NR_033928 plays oncogenic roles in vitro and in vivo

**Fig. 3:**
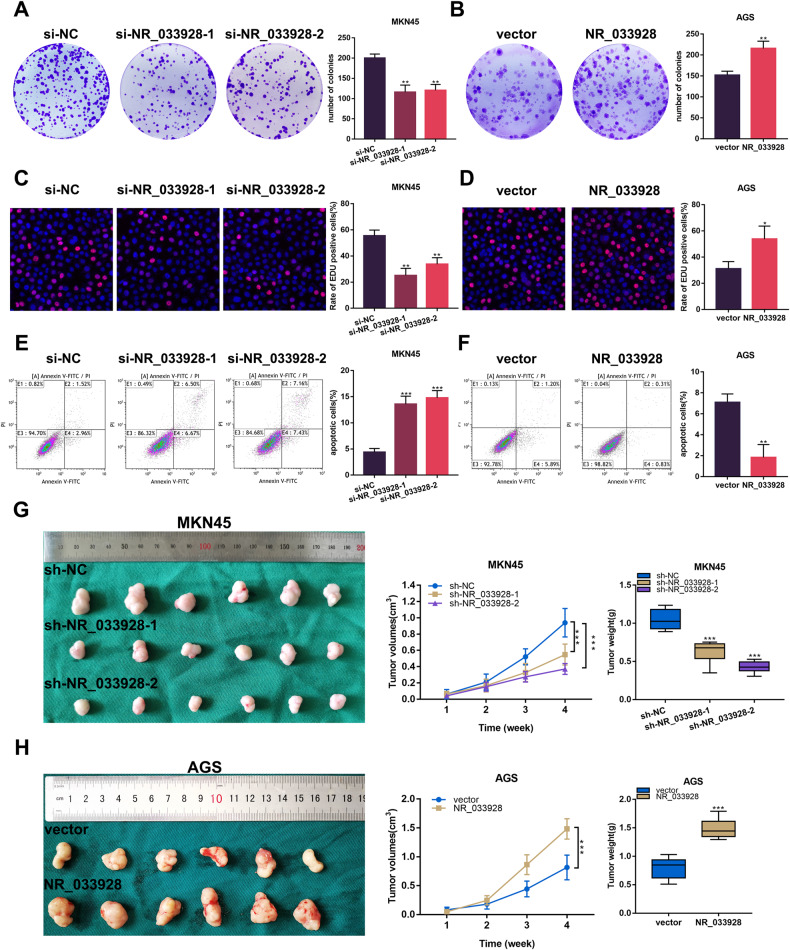
NR_033928 promotes proliferation and inhibits apoptosis in GC. **A**, **B** Colony formation assays in MKN45 cells transfected with si-NR_033928 and AGS cells transfected with NR_033928 overexpressing plasmids. **C**, **D** EDU assays in MKN45 cells transfected with si-NR_033928 and AGS cells transfected with NR_033928 overexpressing plasmids. **E**, **F** Apoptotic assays in MKN45 cells transfected with si-NR_033928 and AGS cells transfected with NR_033928 overexpressing plasmids. **G**, **H** Xenograft tumors comprising MKN45 cells transfected with sh-NR_033928 lentivirus or AGS cells transfected with overexpressing NR_033928 lentivirus. (Graph represents mean ± SD; *p < 0.05, **p < 0.01, and ***p < 0.001).

To investigate the biological effects of NR_033928, gain- and loss-of-function assays were performed in GC cells. NR_033928 siRNAs and overexpressing vectors were transfected into MKN45 and AGS cells separately (Supplementary Fig. S1D, E). Colony formation assays indicated that the knockdown of NR_033928 decreased the colony numbers in MKN45 cells while overexpressing NR_033928 promoted colony formation in AGS cells (Fig. 3A, B). Similarly, EDU assays showed that decreasing NR_033928 expression weakened the proliferative capacity in MKN45 cells and increasing NR_033928 expression strengthened the proliferation ability in AGS cells (Fig. 3C, D). Besides, the flow cytology assays revealed that the ratio of apoptotic cells increased in MKN45 cells transfected with NR_033928 siRNA compared with the control group (Fig. 3E). And the apoptotic cell numbers decreased in AGS cells transfected with NR_033928 overexpressing vectors (Fig. 3F). We further investigated the effect of silencing NR_033928 on tumor proliferation in nude mice. GC cells transfected with NR_033928 shRNAs and overexpressing vectors were injected subcutaneously into 5-week-old Balb/c mice. The xenograft tumor model showed that the tumor weight and volume of mice injected with MKN45-sh-NR_033928 were significantly less than the control group (Fig. 3G). And AGS cells stably transfected with NR_033928 overexpressing lentiviruses promoted the growth of xenograft tumors (Fig. 3H).

Collectively, NR_033928 promoted GC proliferation and inhibited apoptosis both in vitro and in vivo.

### NSUN2 upregulates NR_033928 expression by maintaining its stability in GC

**Fig. 4:**
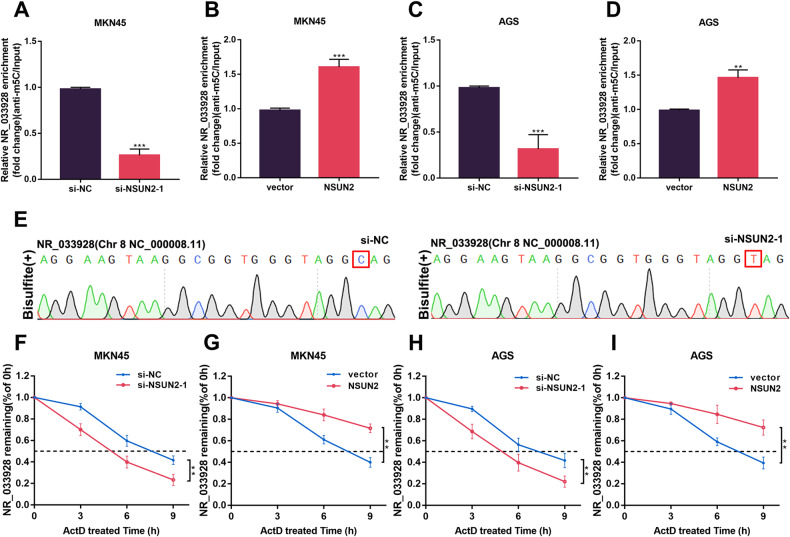
NSUN2 mediates NR_033928 m5C methylation and regulates its expression. **A**–**D** M5C RIP analysis of NR_033928 methylation levels in GC cells transfected with si-NSUN2-1 or NSUN2 overexpressing vectors. **E** Sanger sequencing analysis of m5C sites within NR_033928. **F**, **G** RNA stability analysis of NR_033928 level in MKN45 cells transfected with si-NSUN2-1 or overexpressing NSUN2 vectors after treatment with 5 µg/mL actinomycin D. **H**, **I** RNA stability analysis of NR_033928 level in AGS cells transfected with si-NSUN2-1 or overexpressing NSUN2 vectors after treatment with 5 µg/mL actinomycin D. (Graph represents mean ± SD; **p < 0.01, and ***p < 0.001).

qRT-PCR analysis revealed that the expression of NR_033928 was decreased and increased after transfection of NSUN2 siRNAs and overexpressing vectors in GC cells (Fig. 2D and Supplementary Fig. S1F, G). M5C RIP analysis showed that m5C modification levels of NR_033928 decreased or increased after the transfection of NSUN2 siRNA or overexpressing vectors synchronously in GC cells (Fig. 4A–D). Sanger sequencing of bisulfite-treated NR_033928 validated the specific m5C methylation sites C154 (Fig. 4E). This methylation was completely ablated and base C was converted to base T upon knockdown of NSUN2. Previous studies reported that m5C methylation mediated by NSUN2 affected lncRNA stability [22]. Then we conducted actinomycin D assays to evaluate whether NSUN2 regulated NR_033928 expression through modulating its RNA stability. Results showed that the half-life of NR_033928 was shortened or prolonged in cells transfected with NSUN2 siRNA or overexpressing vectors (Fig. 4F–I).

Taken together, our results demonstrated that NSUN2 catalyzed the m5C modification of NR_033928 and upregulated its expression by enhancing RNA stability.

### NR_033928 regulates GC proliferation and apoptosis through GLS-mediated glutamine metabolism

**Fig. 5:**
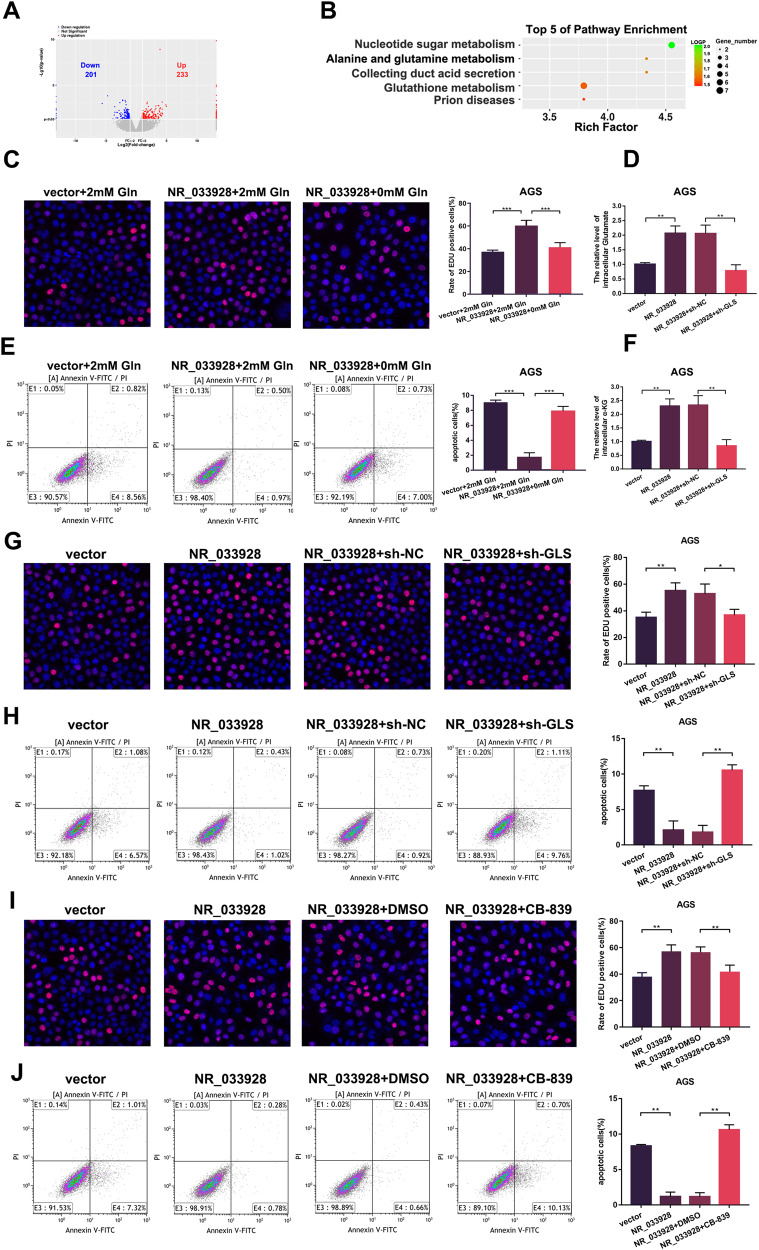
NR_033928 promotes GC progression via GLS-mediated glutamine metabolism. **A** The volcano plot showing the dysregulated genes in NR_033928 knockdown MKN45 cells compared to wild type MKN45 cells. **B** The biological signaling pathways response upon NR_033928 knockdown by Gene KEGG analysis. **C** EDU assays in AGS cells transfected with NR_033928 overexpressing vectors with or without glutamine deletion. **D** Analysis of glutamate concentration in cells transfected with NR_033928 overexpressing vectors or co-transfected with sh-GLS lentivirus. **E** Apoptotic assays in AGS cells transfected with NR_033928 overexpressing vectors with or without glutamine deletion. **F** Analysis of α-KG concentration in cells transfected with NR_033928 overexpressing vectors or co-transfected with sh-GLS lentivirus. **G** EDU assays in AGS cells transfected with NR_033928 overexpressing vectors or co-transfected with sh-GLS lentivirus. **H** Apoptotic assays in AGS cells transfected with NR_033928 overexpressing vectors or co-treated with sh-GLS lentivirus. **I** EDU assays in AGS cells transfected with NR_033928 overexpressing vectors or co-treated with CB-839 (1 μm 72 h). **J** Apoptotic assays in AGS cells transfected with NR_033928 overexpressing vectors or co-treated with CB-839 (1 μm 72 h). (Graph represents mean ± SD; *p < 0.05, **p < 0.01 and ***p < 0.001).

To investigate the underlying molecular mechanisms by which NR_033928 regulated GC proliferation and apoptosis, RNA-seq was performed in NR_033928 deficient and wild-type cells (Fig. 5A). Through KEGG analysis, we noticed that the NR_033928 expression was positively relative to the glutamine metabolism pathway, which was closely related to cancer progression (Fig. 5B). EDU assays indicated that the proliferation ability was enhanced and attenuated in NR_033928 overexpressing cells cultured in normal medium and glutamine deprivation medium (Fig. 5C). Apoptosis assays showed that the proportion of apoptotic cells decreased in NR_033928 overexpressing cells cultured in normal medium and was rescued in glutamine depleted medium (Fig. 5E). Among all the differently expressed genes, GLS is a key gene participating in glutamine decomposition. Western blot and PCR results indicated that NR_033928 regulated GLS expression (Supplementary Fig. S2A, B). Besides, our previous studies also found that circLMO7 promoted GC proliferation, migration, and invasion through GLS glutamine metabolism [25]. Whether NR_033928 regulated GC malignant biological behaviors through GLS-mediated glutamine metabolism remains further investigated.

First, we found that overexpressing NR_033928 upregulated the glutamate and α-KG content while co-transfection of sh-GLS downregulated their content in GC cells (Fig. 5D, F). Then, EDU assays showed the proportion of EDU positive cells was increased by overexpressing NR_033928 and decreased when NR_033928 overexpressing vectors and sh-GLS were co-transfected in AGS cells (Fig. 5G). EDU assays showed that the inhibitory effect of NR_033928 siRNA on proliferation in MKN45 cells was rescued by overexpressing GLS (Supplementary Fig. S2C). Then apoptosis assays showed that the proportion of apoptotic cells was reduced by overexpressing NR_033928 and rescued by co-transfecting NR_033928 overexpressing vectors and sh-GLS in AGS cells (Fig. 5H). Besides, the proportion of apoptotic cells was increased by transfection of NR_033928 siRNAs and reduced by co-transfection of GLS overexpressing vectors (Supplementary Fig. S2D). Telaglenastat (CB-839), a first-in-class and selective inhibitor of GLS, was also applied in AGS cells. The EDU assays showed that CB-839 reduced the proliferative activity in cells transfected with NR_033928 overexpressing vectors in AGS cells (Fig. 5I). Apoptosis assays indicated that CB-839 promoted apoptosis in cells transfected with NR_033928 overexpressing vectors in AGS cells (Fig. 5J).

These results indicated that NR_033928 exerted its function in GC through GLS mediated glutamine metabolism.

### NR_033928 interacts with IGF2BP3/HUR complex and promotes its formation

NR_033928 was found to regulate GLS expression (Supplementary Fig. S2A, B). Considering the main cytoplasmic distribution of NR_033928, we further explored the potential mechanism by which NR_033928 activates GLS expression post-transcriptionally. Online database (http://bio-bigdata.hrbmu.edu.cn/LncACTdb/) prediction results showed that there were no miRNA response elements by which NR_033928 regulated GLS expression.

**Fig. 6:**
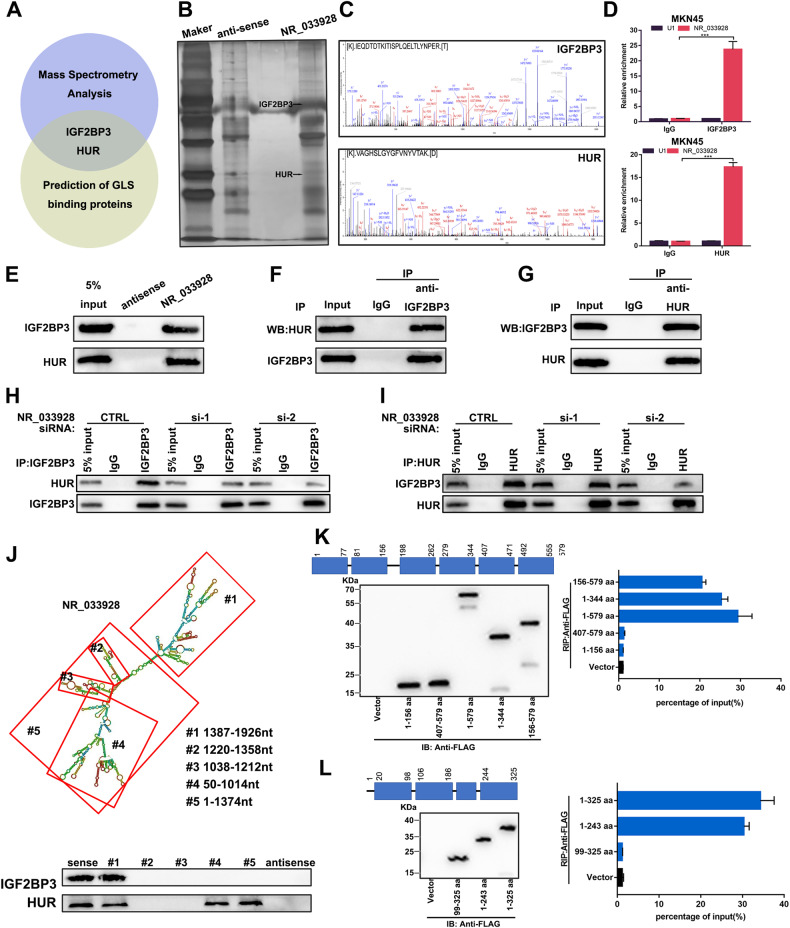
NR_033928 directly binds to IGF2BP3/HUR complex. **A** A Venn diagram showing interested proteins analyzed by mass spectrometry and prediction of GLS binding proteins. **B**, **C** Mass spectrometry analysis of two specific bands (arrows). **D** NR_033928 was immunoprecipitated with anti-IGF2BP3 and anti-HUR. qRT-PCR showed the relative expression of NR_033928. **E** RNA pull-down assays showed that IGF2BP3 and HUR were immunoprecipitated by NR_033928 probes. **F**, **G** Interactions between HUR and IGF2BP3 were verified by co-immunoprecipitation assays. **H**, **I** Co-immunoprecipitation assays were conducted to assess the interactions between HUR and IGF2BP3 in cells transfected with NR_033928 siRNAs. **J** IGF2BP3 and HUR were detected with sequentially deleted NR_033928 fragments by RNA pull-down. **K** RIP assays of NR_033928 bound by truncated IGF2BP3 mutants. Left upper panel: the schematic structures showing IGF2BP3 protein domains. Left lower panel: Immunoblot of the FLAG-IGF2BP3 mutants. Right panel: RIP analysis of NR_033928 enrichment in 293 T cells transfected with truncated IGF2BP3 mutants. **L** RIP assay of NR_033928 bound by truncated HUR mutants. Left upper panel: the schematic structures showing HUR protein domains. Left lower panel: immunoblot of the FLAG-HUR mutants. Right panel: RIP analysis of NR_033928 enrichment in 293 T cells transfected with truncated HUR mutants. (Graph represents mean ± SD; ***p < 0.001).

Many studies demonstrated that lncRNAs could exert their functions by interacting with a variety of proteins in cancer [26, 27]. To identify functional proteins interacting with NR_033928, RNA pull-down and mass spectrometry analysis were performed three times independently in GC cells. Ten potential protein partners were finally filtered out based on unique peptide number >20 and only in all three independent experiments (Supplementary Table S1). Among all the candidate proteins, IGF2BP3 and HUR attracted our attention, which existed in both mass spectrometry results and predicated GLS binding proteins results (http://starbase.sysu.edu.cn/) (Fig. 6A–C). Besides, IGF2BP3 and HUR were proved to regulate RNA stability [28, 29]. The actinomycin D assay showed that NR_033928 enhanced GLS mRNA stability (Supplementary Fig. S2E). Next, RNA immunoprecipitation assays were performed using IGF2BP3 and HUR antibodies. Results showed that the expression of NR_033928 was significantly higher in IGF2BP3 or HUR antibody group compared to the IgG group (Fig. 6D). RNA pull-down analysis verified that recombinant IGF2BP3 and HUR could be precipitated by biotin-labeled NR_033928 (Fig. 6E). We put forward a hypothesis that NR_033928 acted as a scaffold of the IGF2BP3/HUR complex to promote GLS mRNA stability in GC.

Co-immunoprecipitation assays showed that IGF2BP3 and HUR interacted with each other in GC (Fig. 6F, G). Silencing NR_033928 had no effect on the protein expression of IGF2BP3 and HUR (Supplementary Fig. S2F). Furthermore, silencing NR_033928 through siRNAs reduced the protein amount of HUR or IGF2BP3 precipitated by IGF2BP3 or HUR antibodies (Fig. 6H, I). RNAfold (http://rna.tbi.univie.ac.at/) and RNA structure (http://rna.urmc.rochester.edu/RNAstructure) were applied to predict the secondary structure of NR_033928. To further clarify how NR_033928 recruits IGF2BP3 and HUR, several NR_033928 deletion mutants were designed. RNA pull-down analysis showed that NR_033928 #1 fragment containing 1387–1926nt interacted with IGF2BP3 and #1, #4, and #5 fragments containing 1387–1926nt, 50–1014nt, and 1–1374nt interacted with HUR (Fig. 6J). In addition, a serious of FLAG-tagged truncated IGF2BP3 and HUR were constructed based on their intrinsic protein domains. RIP assays demonstrated that the IGF2BP3 domain (198-344aa) and HUR domain (1-99aa) specifically interacted with NR_033928 (Fig. 6K, L).

In all, NR_033928 interacted with IGF2BP3/HUR complex and facilitated its formation.

### NR_033928 promotes interactions between IGF2BP3/HUR complex and GLS mRNA

IGF2BP3/HUR complex had been proved to stabilize s series of oncogenic mRNAs [30, 31]. Thus, we wanted to investigate whether NR_033928 regulated the stability of GLS through acting as a scaffold for IGF2BP3/HUR complex and GLS mRNA.

**Fig. 7:**
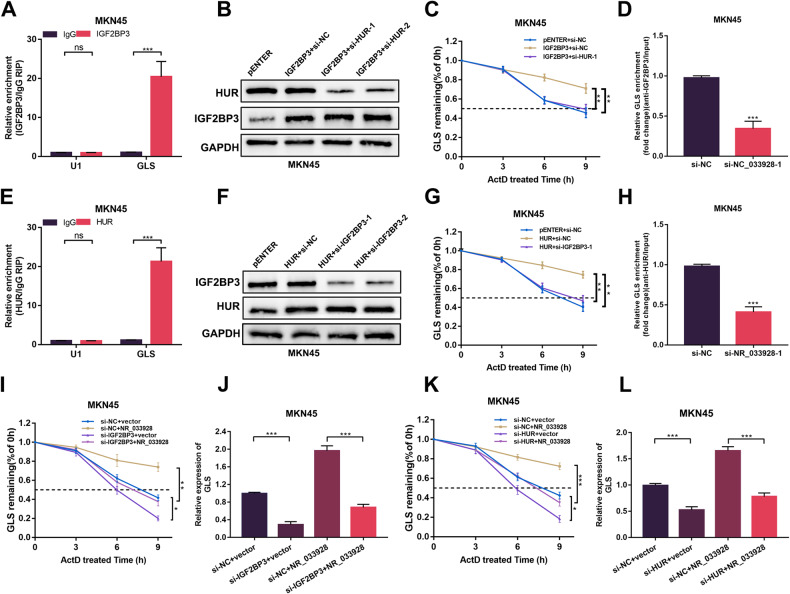
NR_033928 acts as a scaffold between IGF2BP3/HUR and GLS. **A**, **E** RIP assays of relative GLS levels bound by IGF2BP3 antibody or HUR antibody. **B** Western blot analysis of transfection of IGF2BP3 overexpression vectors and si-HUR in MKN45 cells. **C** RNA stability analysis of GLS levels in MKN45 cells transfected with IGF2BP3 overexpression vectors and si-HUR after treatment with 5 µg/mL actinomycin D. **D**, **H** RIP assays of GLS bound by IGF2BP3 or HUR antibody in MKN45 cells transfected with NR_033928 siRNAs. qRT-PCR was used to detect the GLS expression. **F** Western blot analysis of transfection of HUR overexpression vectors and si-IGF2BP3 in MKN45 cells. **G** RNA stability analysis of GLS levels in MKN45 cells transfected with HUR overexpression vectors and si-IGF2BP3 after treatment with 5 µg/mL actinomycin D. **I** RNA stability analysis of GLS levels in MKN45 cells transfected with si-IGF2BP3 or co-transfected with NR_033928 overexpression vectors after treatment with 5 µg/mL actinomycin D. **J** qRT-PCR analysis of GLS expression in MKN45 cells transfected with si-IGF2BP3 or co-transfected with NR_033928 overexpression vectors. **K** RNA stability analysis of GLS levels in MKN45 cells transfected with si-HUR or co-transfected with NR_033928 overexpression vectors after treatment with 5 µg/mL actinomycin D. **L** qRT-PCR analysis of GLS expression in MKN45 cells transfected with si-HUR or co-transfected with NR_033928 overexpression vectors. (Graph represents mean ± SD; *p < 0.05, **p < 0.01 and ***p < 0.001).

First, RIP assays implied that HUR and IGF2BP3 bound to GLS (Fig. 7A, E and Supplementary Fig. S3A, E). Then, western blot analysis confirmed that overexpressing plasmids and siRNAs of IGF2BP3 and HUR were constructed and transfected in GC cells (Fig. 7B, F and Supplementary Fig. S3B, F). Actinomycin D assays showed that the half-life of GLS was prolonged with overexpressing IGF2BP3 and shortened with co-transfection with HUR siRNA (Fig. 7C and Supplementary Fig. S3C). Similar results were detected in actinomycin D assays using HUR overexpressing vectors and IGF2BP3 siRNAs (Fig. 7G and Supplementary Fig. S3G). These results showed that IGF2BP3 and HUR coordinated to enhance GLS stability.

RIP assays also showed that silencing NR_033928 or overexpressing NR_033928 decreased or increased the amount of GLS bound by IGF2BP3 and HUR (Fig. 7D, H and Supplementary Fig. S3D, H). Actinomycin D assays showed that the half-life of GLS was shortened with si-IGF2BP3 or si-HUR and prolonged with co-transfection with NR_033928 overexpressing vectors (Fig. 7I, K and Supplementary Fig. S3I, K). Besides, qRT-PCR analysis of GLS expression showed that inhibiting IGF2BP3 expression decreased the expression of GLS that was increased by overexpressing NR_033928 (Fig. 7J and Supplementary Fig. S3J). Similarly, inhibiting HUR expression decreased the expression of GLS which was increased by overexpressing NR_033928 (Fig. 7L and and Supplementary Fig. S3L).

Thus, NR_033928 regulates GLS expression through modulating GLS stability by acting as a scaffold for IGF2BP3/HUR complex and GLS.

### α-KG promotes NR_033928 promoter demethylation which in turn increases NR_033928 expression

**Fig. 8:**
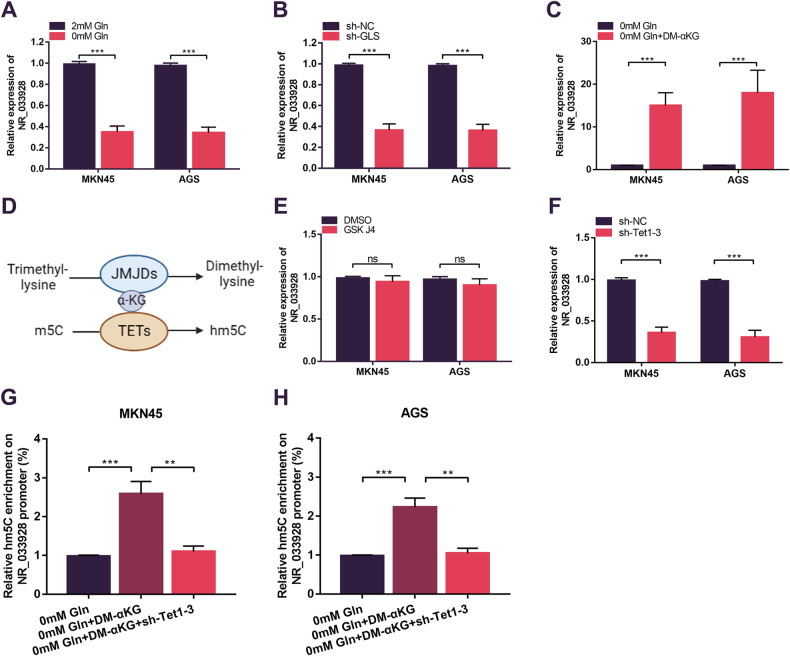
α-KG upregulates NR_033928 expression by promoting NR_03928 promoter demethylation. **A** qRT-PCR analysis of NR_033928 expression in glutamine depletion medium in GC cells. **B** qRT-PCR analysis of NR_033928 expression in GC cells transfected with sh-GLS. **C** qRT-PCR analysis of NR_033928 expression in glutamine depletion medium in GC cells after DM-α-KG(10 µM 24h) treatment. **D** Schematic diagram of α-KG-dependent histone demethylation by JMJDs and DNA demethylation by TETs. **E** qRT-PCR analysis of NR_033928 expression in GC cells after GSK J4(4 µM 24h) treatment. **F** qRT-PCR analysis of NR_033928 expression in GC cells upon combined silencing of Tet1, Tet2 and Tet3. **G**, **H** hMeDIP assays of the relative hm5C methylation levels of NR_033928 promoter in GC cells in glutamine depletion medium treated with DM-α-KG(10 µM 24h) or combined with sh-Tet1-3. (Graph represents mean ± SD; **p < 0.01 and ***p < 0.001).

Interestingly, we found that the expression of NR_033928 was significantly decreased in glutamine-depletion medium (Fig. 8A). And silencing GLS also decreased NR_033928 expression (Fig. 8B). Then we explored whether glutamine downstream metabolites affected NR_033928 expression. qRT-PCR analysis indicated that NR_033928 expression was increased by adding exogenous synthetic α-KG (DM-α-KG) instead of other metabolites of glutamine (Fig. 8C and Supplementary Fig. S4A–F).

Abnormal accumulation of α-KG plays an important role in the genome epigenetic regulation by acting as a co-factor of DNA demethylases (TETs) and histone demethylases (JMJDs) (Fig. 8D) [32]. We put forward a hypothesis that increased α-KG might increase the expression of NR_033928 by promoting DNA/ histone demethylation. Inhibiting histone demethylases (JMJDs) by GSK-J4 did not alter the expression of NR_033928 (Fig. 8E). TET proteins promote DNA demethylation by catalyzing the oxidation of 5-methylcytosine (m5C) to 5-hydroxymethylcytosine (hm5C) [33]. The combined silencing of the Tet family (Tet1 Tet2 and Tet3) significantly decreased the expression of NR_033928 (Fig. 8F and Supplementary Fig. S4G, H). Bioinformatics analysis indicated that there were several CpG islands in the NR_033928 promoter. hMeDIP assays indicated that the hm5C level of NR_033928 promoter was increased by adding exogenous DM-αKG and decreased upon transfection of sh-Tet1-3 in cells cultured in glutamine-depletion medium (Fig. 8G, H).

Collectively, α-KG promoted NR_033928 expression in TETs-dependent DNA demethylation manners.

### Clinical relevance of NR_033928 in GC

**Fig. 9:**
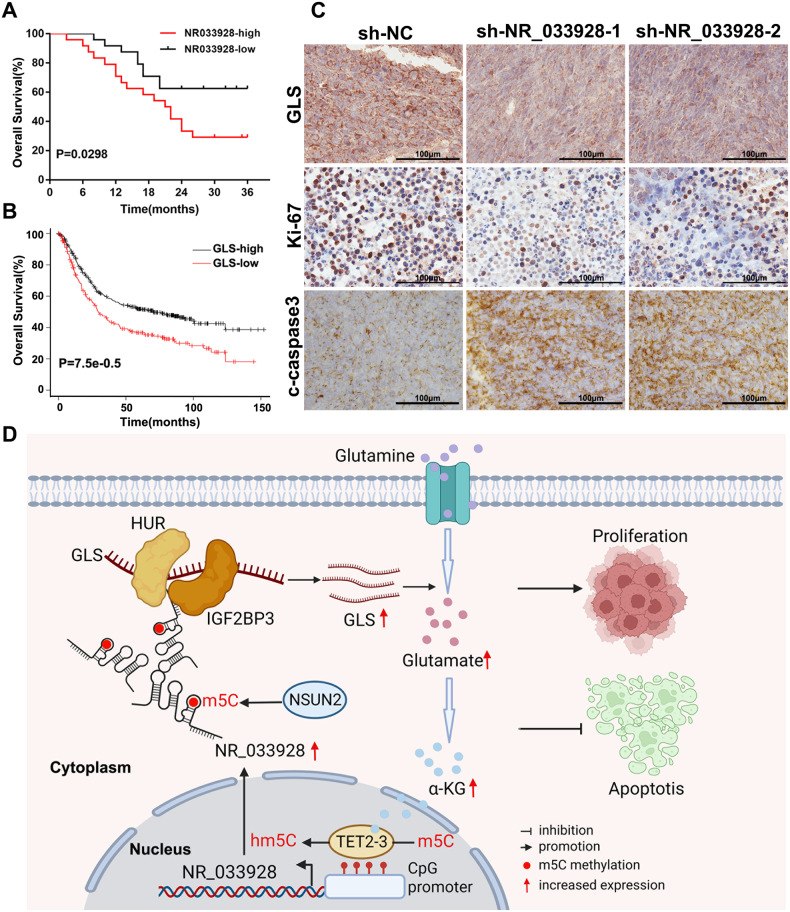
NR_033928 serves as a prognostic and therapeutic biomarker in GC. **A** Kaplan–Meier analysis of the relation between NR_033928 expression and patients’ survival. **B** Kaplan–Meier analysis of the relation between GLS expression and patients’ survival. **C** Representative images of immunohistochemistry analysis of GLS, Ki-67 and c-caspase3 expression in xenografts transfected with sh-NR_033928 lentivirus. Scale bar = 100 μm. **D** A schematic diagram of NR_033928 functions in GC. NSUN2 mediated NR_033928 m5C methylation thereby improving NR_033928 expression. NR_033928 promoted GC progression by glutamine metabolic reprogramming. Mechanistically, NR_033928 functions as a scaffold to recruit HUR and IGF2BP3 and increased GLS expression.

Kaplan–Meier analysis of follow-up data in our center indicated that the expression of NR_033928 had a negative correlation with patients’ overall survival (Fig. 9A). Besides, results from the online Kaplan–Meier model (http://kmplot.com/analysis/) showed that patients with high expression of GLS had lower overall survival than those with low GLS expression (Fig. 9B).

In the xenograft tumor model, all mice were sacrificed in the fourth week and tumors were used for immunohistochemistry analysis (Fig. 9C). Immunohistochemistry staining of GLS indicated that knockdown of NR_033928 significantly reduced the expression of GLS. Besides, analysis of the proliferation marker ki67 and apoptosis marker c-caspase3 indicated that silencing NR_033928 decreased the expression of ki-67 and enhanced the expression of c-caspase3.

Thus, NR_033928 could serve as a potential prognostic marker and silencing NR_033928 could inhibit tumor proliferation and promote apoptosis in GC.

## Discussion

With the development of high-throughput sequencing, more and more lncRNAs are identified in mammalian cells. LncRNA was originally thought to be a byproduct of transcription without any functions. As the research moved along, accumulating evidence indicated that lncRNAs played important roles in various biological processes of normal tissue and tumors [13]. Many studies showed that lncRNAs impacted GC carcinogenesis, progression, and chemoresistance [26, 34, 35]. Our previous also proved that lncRNA TRPM2-AS attenuates GC proliferation, migration, and invasion [36].

Aberrant epigenetic modifications, including DNA modifications, histone modifications, and RNA modifications, contribute to tumor progression [37]. In recent years, studies on RNA modifications such as m6A, m5C, and m1A RNA modifications become hotspots in cancer research [34]. Till now, a majority of studies concentrated on the roles of m6A and m1A in cancer. Little research was conducted on m5C modifications. m5C modification was first reported in transfer RNAs and ribosomal RNAs [38]. With the advance in m5C detection techniques, more than ten thousand m5C modifications were identified in transcriptomes by using bisulfite sequencing, m5C RNA immunoprecipitation sequencing (m5C-RIP-seq), and so on. And m5C was found to exist not only in tRNAs, rRNAs, and mRNAs but also in lncRNAs and other RNA species [39]. Most research focused on mRNA m5C modifications in cancer. Little studies were performed to investigate the effect of m5C modifications on lncRNAs.

In this study, we performed m5C-RIP-seq in 3 pairs of GC tissues and their matched normal tissues. Through combined screening, we identified a hypermethylated lncRNA, NR_033928, which was significantly upregulated in GC cells and tissues. NR_033928 was previously reported in pancreatic ductal adenocarcinoma (PDAC), which was highly expressed in PDAC and promoted PDAC proliferation, migration, and invasion, and suppressed apoptosis [40]. Function assays indicated that NR_033928 promoted GC growth and suppressed apoptosis in our study. However, NR_033928 had no effect on GC migration and invasion. We hypothesize that the different effect of NR_033928 on tumor malignant behaviors is due to different genetic backgrounds, which needs further investigation.

NR_033928 was identified as a lncRNA with high m5C modification. In our results, we further explored the mechanism by which m5C modification impacted NR_033928. Sanger sequencing revealed that m5C methyltransferase NSUN2 promoted NR_033928 m5C methylation and specifically methylated C154. Actinomycin D assays indicated that NSUN2 promoted the stability of NR_033928 and upregulated its expression. Except for RNA modifications, there are other factors that may impact the expression of lncRNA, such as DNA methylation or histone modification, which deserves further exploration.

To find the downstream of NR_033928, next-generation sequencing was conducted in cells stably transfected with NR_0333928 siRNA and the control group. Through Kyoto Encyclopedia of Genes and Genomes analysis, we found NR_033928 was positively related to glutamine metabolism. Dysregulation of glutamine metabolism widely exists in cancer to support rapid proliferation [17]. And qRT-PCR and western blot results indicated that GLS was the downstream of NR_033928. GLS gene encodes kidney-type glutaminase, which catalyzes the conversion of glutamine to glutamate, the first key reaction of glutaminolysis. The GLS enzyme is usually upregulated in various cancer and promotes cancer cell proliferation and suppresses apoptosis, including breast cancer, lung cancer, colon cancer, and so on [41, 42]. There are many studies reporting that GLS could be regulated in a variety of mechanisms. For example, SUCLA2 mediated GLS succinylation and promoted tumor cell progression [41]. GSK3 signaling axis regulated GLS in lung cancer [43]. In this study, we first reported that GLS could be regulated by NR_033928 in GC. Rescue experiments proved that NR_033928 promoted GC malignant behaviors through GLS-mediated glutamine metabolism.

Interacting with RNA binding proteins is the important way by which lncRNAs exert their functions [44]. FISH assays indicated that NR_033928 was mainly localized in the cytoplasm. RNA pull-down and mass spectrometry were applied to identify potential protein partners in three independent experiments. Through scanning the results of mass spectrometry, we screened ten potential proteins with high confidence which existed in NR_033928 sense group and were absent in antisense group. Previous studies reported that lncRNAs could function as a scaffold by interacting with two or more proteins. For example, GClnc1 acted as a scaffold of WDR5 and KAT2A to promote GC progression [26]. SLC26A4-AS1 suppressed thyroid cancer metastasis by acting as a scaffold of DDX5 and TRIM25 [45]. Combined analysis of mass spectrometry results and prediction results of GLS binding proteins, IGF2BP3 and HUR were finally identified as interacting proteins. The two proteins were previously reported to promote mRNA stability. For example, IGF2BP3 was found to promote the stability of HMGA2 [46]. HUR was proved to stabilize uPA and uPAR mRNAs [47]. Moreover, IGF2BP3 and HUR formed a complex to promote tumorigenicity by stabilizing oncogenic transcripts [30]. RNA pull-down and COIP assays proved that NR_033928 interacted with IGF2BP3/HUR complex and promoted the formation of the complex. Moreover, we further identified the specific RNA sequence and protein domain that mediated the interaction of NR_033928 and IGF2BP3 or HUR. Next, RIP, actinomycin D, and qRT-PCR indicated that IGF2BP3 and HUR bound to GLS and promoted the stability of GLS. Knockdown of NR_033928 weakened the effect of the IGF2BP3/HUR complex on GLS stability.

Metabolic reprogramming has been proved to participate in various biological processes and regulatory networks in cancers. Interestingly, we found that the downstream metabolite of glutamine, α-KG, could increase NR_033928 expression in the study. qRT-PCR and hMeDIP assays proved that accumulation of α-KG upregulated NR_033928 expression by enhancing TETs-dependent NR_033928 promoter demethylation. Hereto, we found that the expression of NR_033928 was regulated synergistically by RNA m5C methylation mediated by NSUN2 and DNA hm5C demethylation mediated by TETs. This shed new light on how lncRNAs are regulated in cancers.

We also investigate the clinical value of NR_033928 in this study. Kaplan–Meier analysis indicated that high expression of NR_033928 and GLS was positively correlated with poor prognosis of GC patients. IHC analysis of xenograft in mice showed that silencing NR_033928 significantly decreased the GLS and Ki-67 expression and increased the expression of c-caspase3. This indicated that NR_033928 could serve as a prognostic biomarker and therapeutic target in GC.

Collectively, we found that NSUN2 was highly expressed in GC cells and tissues and associated with poor prognosis in GC. Further analysis indicated that NSUN2 maintained NR_033928 stability in an m5C-dependent manner and upregulated its expression. NR_033928 promoted GC proliferation and suppressed apoptosis by increasing GLS expression. NR_033928 acted as a scaffold of the IGF2BP3/HUR complex and GLS. α-KG could increase NR_033928 expression by enhancing its promoter demethylation, thereby forming a positive feedback loop. Moreover, high NR_033928 expression was associated with patients’ prognosis. Our results highlighted that NR_033928 may serve as a therapeutic target for RNA interference strategies and biomarker of prognosis in GC.

## Methods

### Patients and specimens

A total of 51 pairs of GC and para-cancerous specimens were collected from the first affiliated hospital of Nanjing Medical University. This study was approved by the ethics committee of the hospital. These samples were gathered from GC patients in 2019–2022. All samples were collected immediately after radical GC resection and kept in liquid nitrogen. Informed consent was provided by all participants.

### Cell lines and cell culture

GES-1, HGC-27, MKN28, MKN45, AGS, SNU1 and HEK-293 T cell lines were purchased from ATCC, the Cell Center of Shanghai Institutes for Biological Sciences. GES-1, HGC-27, MKN28, MKN45, and SNU1 cells were cultured in 1640 medium. AGS cells were cultured in F12K medium. HEK-293 T cells were cultured in DMEM medium. All medium were supplemented with 10% fetal bovine serum and 1% penicillin/streptomycin. All cells were incubated in an incubator with constant 37 °C and 5% CO_2_.

### Bioinformatic analysis

The public data TCGA-STAD was downloaded from The Cancer Genome Atlas (TCGA) database (https://xenabrowser.net/datapages/), including 375 gastric cancers and 32 normal tissues. The “limma” R package was used to analyze the differentially expressed genes, and “ggplot2” and “RCircos” were employed to visualize. We perform KEGG enrichment analysis by DAVID Bioinformatics Resources (https://david.ncifcrf.gov/). Overall survival was calculated using Kaplan–Meier model (http://kmplot.com/analysis/). Several online databases were used to predict the function of NR_033928, including CPC2 (http://cpc2.gao-lab.org/), CPAT (http://rna-cpat.sourceforge.net/), LncACTdb 3.0 (http://bio-bigdata.hrbmu.edu.cn/LncACTdb/), starBase v2.0 (http://starbase.sysu.edu.cn/), ViennaRNA Web Services (http://rna.tbi.univie.ac.at/), and RNAstructure (http://rna.urmc.rochester.edu/RNAstructure).

### m5C MeRIP sequencing and MeRIP-PCR

Briefly, m5C RNA MeRIP-seq was performed using a GenSeq® m5C MeRIP (Cloud-Seq Inc, China). The input samples with or without m5C immunoprecipitation samples were used for RNA-seq library generation with the NEBNext® Ultra II Directional RNA Library Prep Kit (New England Biolabs Inc., USA). cDNA library sequencing was performed on an Illumina HiSeq4000. For the m5C-RIP-qPCR, the RNA enrichment was obtained from the IP beads and was analyzed by qPCR [48].

### LncRNA profiling and mRNA next-generation sequencing

Total RNA of GC cells was extracted and purified using TRIzol reagent (Invitrogen, CA, USA) under the instructions of the manufacturer’s recommendations. lncRNA expression profiles were investigated using SBC Human lncRNA Microarray. Agilent Feature Extraction software was used to extract the raw data. The Quantile algorithm, Gene Spring (Agilent Technologies) was used for microarray statistics analysis.

Total RNA was isolated from wild MKN45 cells and si-NR_033928 MKN45 cells, each with three replicates. mRNA expression profiling on total RNA was performed by GENEWIZ. Next-generation sequencing libraries were constructed using the NEBNextR Ultra™ RNA Library Prep Kit for Illumina® and sequenced on an Illumina HiSeq instrument according to the manufacturer’s instructions (Illumina).

### Oligonucleotide, lentiviral, and plasmid transfection

LncRNA and mRNA siRNAs were purchased from RiboBio (Guangzhou, China). The lentiviral targeting lncRNA and mRNA were generated by GenePharma (Shanghai, China). Overexpressing plasmids were purchased from WZ Biosciences (Shandong, China). All cells were transfected with siRNAs and plasmids by using Lipo3000 under the product instructions. The detailed sequences are listed in Supplementary Table S2.

### RNA extraction and PCR

The RNA extraction and PCR were performed as described previously [49]. Primers used in this study are listed in Supplementary Table S2.

### Fluorescence in situ hybridization

Fluorescence in situ hybridization (FISH) was performed as described previously [49]. Fam-labeled oligo(dT)50 probes were purchased from Ribobio (Guangzhou, China). The probe for FISH was listed in Supplementary Table S4.

### Western blotting and antibodies

Western blotting (WB) was performed as described previously [49]. Antibodies used in this study are listed in Supplementary Table S3.

### RNA half-life measurement

The stability of NR_033928 was assessed by additionally adding actinomycin D(5 μg/ml) into the cell medium at the indicated time. Then total RNA was used for RT-PCR to calculate the half-life of NR_033928.

### Colony formation assay

The colony formation assay was performed as described previously [49].

### 5-Ethynyl-2′-deoxyuridine assay

The 5-Ethynyl-2′-deoxyuridine assay (EDU) was performed with a Cell-Light EDU Cell Proliferation Kit (RiboBio) as described previously [50].

### Apoptosis assay

The apoptosis assay was performed through Annexin V and PI Apoptosis Kit (UE, China) in the Cytoflex flow cytometer (Beckman, USA), as described previously [49].

### The detection of glutamate and α-Ketoglutarate

A Glutamate Assay Kit and alpha-Ketoglutarate Assay Kit were purchased from Abcam (ab138883, USA) and Sigma (MAK054, USA). The detection was performed under the instructions of product manuals.

### Chemical reagents

CB-839 (HY-12248), GSK J4 (HY-15648B), and isocitrate (HY-W009362) were purchased from MCE (USA). Fumarate (47910), succinate (S9512), citrate (C0759), diethyl malate (7554-12-3) and diethyl-ester OAA (40876-98-0) purchased from Sigma-Aldrich (USA). All reagents were dissolved to the indicated concentration in dimethyl sulfoxide.

### Hydroxymethylated DNA immunoprecipitation

The hydroxymethylated DNA immunoprecipitation(hMeDIP) was performed by using EpiQuik™ hydroxymethylated DNA immunoprecipitation kit (EpiGentek, USA). Briefly, the DNA was exacted and sonicated to 200–1000 bp fragments by ultrasonic shredding. 5-hmC antibody and IgG were added to DNA separately. Next, the purified DNA was obtained by washing, release and elution of DNA. Then the purified products were analyzed by qPCR. The primer sequences used were shown in Supplementary Table S2.

### Nude mice experiments

The xenograft tumor model was performed as described previously [50]. The tumor weight and volume were used to assess the effect of NR_033928 on tumor proliferation.

### Immunohistochemistry

Immunohistochemistry (IHC) analysis was performed as described previously [50]. Indicated antibodies are listed in the Supplementary Table S3.

### RNA binding protein immunoprecipitation assay

The RNA Binding Protein Immunoprecipitation Assay (RIP) assay was performed as described previously [51]. Imprint® RNA Immunoprecipitation Kit (Sigma-Aldrich, USA), anti-IGF2BP3 and anti-HUR were purchased to perform the RIP assay following the instruction manuals. Indicated recombinant proteins are listed in Supplementary Table S3.

### Co-immunoprecipitation assay

The Co-immunoprecipitation assay (CO-IP) assay was performed as described previously by using anti-IGF2BP3 and anti-HUR under the instructions of Pierce Co-Immunoprecipitation Kit (Thermo Scientific, USA) protocols [51].

### RNA pull-down and mass spectrometry

RNA pull-down and mass spectrometry were performed as described previously [51]. Pure-Binding^TM^ RNA-Protein pull-down Kit (Geneseed) was purchased for RNA pull-down assay. The bio-labeled RNA and protein complex was used for western blotting and mass spectrometry analysis. The probe for RNA pull-down was listed in Supplementary Table S4.

### Sanger sequencing of PCR products

For the validation of NR_033928 methylated sites, bisulfite converted RNA was reverse transcribed into cDNA using the PrimeScript™ II 1st Strand cDNA Synthesis Kit (Takara, Japan) according to the manufacturer’s instructions. cDNA was amplified by PCR using specific primers for bisulfite-treated RNAs and the PyroMark PCR Kit (Qiagen, Germany). Then PCR products were used for Sanger sequencing.

### Statistics analysis

All statistical analyses were carried out using the GraphPad Prism 7.0 or SPSS (SPSS Inc., Chicago, USA). One-Way ANOVA test and the Student’s *t* test were used to test the difference in most of experiments. The chi-square test was used to analyze the association between the expression of NR_033928 and clinicopathological parameters. Overall survival was calculated using Kaplan–Meier model (http://kmplot.com/analysis/). Differences were considered significant with a value of *p* < 0.05.

### Reporting summary

Further information on research design is available in the Nature Research Reporting Summary linked to this article.

## Supplementary information

Original Data Files

supplementary materials

Reporting Summary

## Data Availability

All data supporting this study are present in the paper and Supplementary Materials.
