# Supplementary material for: m5C-methylated lncRNA NR_033928 promotes gastric cancer proliferation by stabilizing GLS mRNA to promote glutamine metabolism reprogramming
Source: Cell Death Dis. 2023 Aug 15;14(8):520. doi: 10.1038/s41419-023-06049-8 (PMC10427642; doi:10.1038/s41419-023-06049-8)
Supplement: Supplementary file 1 — Original Data Files [file 41419_2023_6049_MOESM1_ESM.docx]

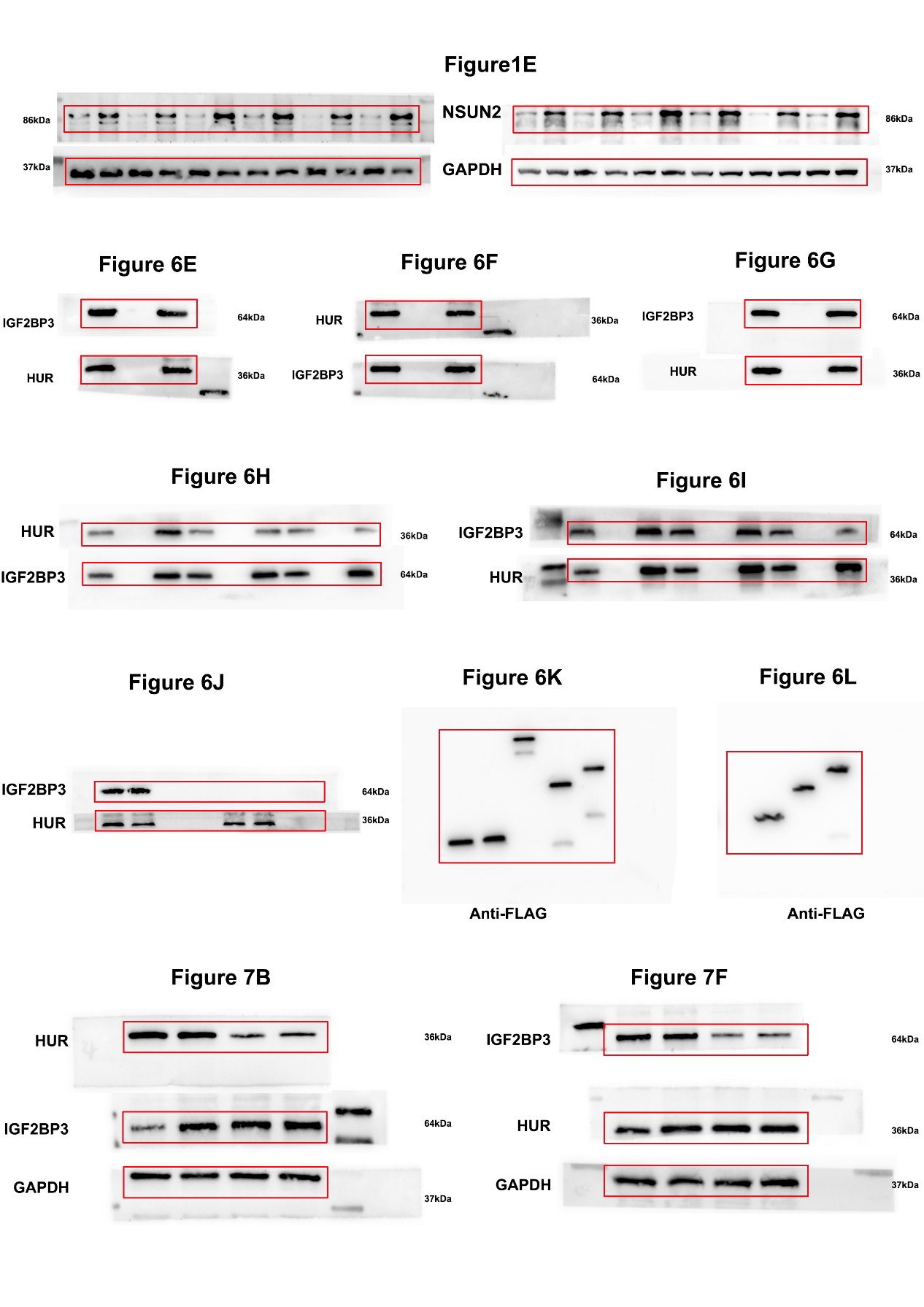


The uncropped scans of western blots and gels from the figures of the main manuscript.


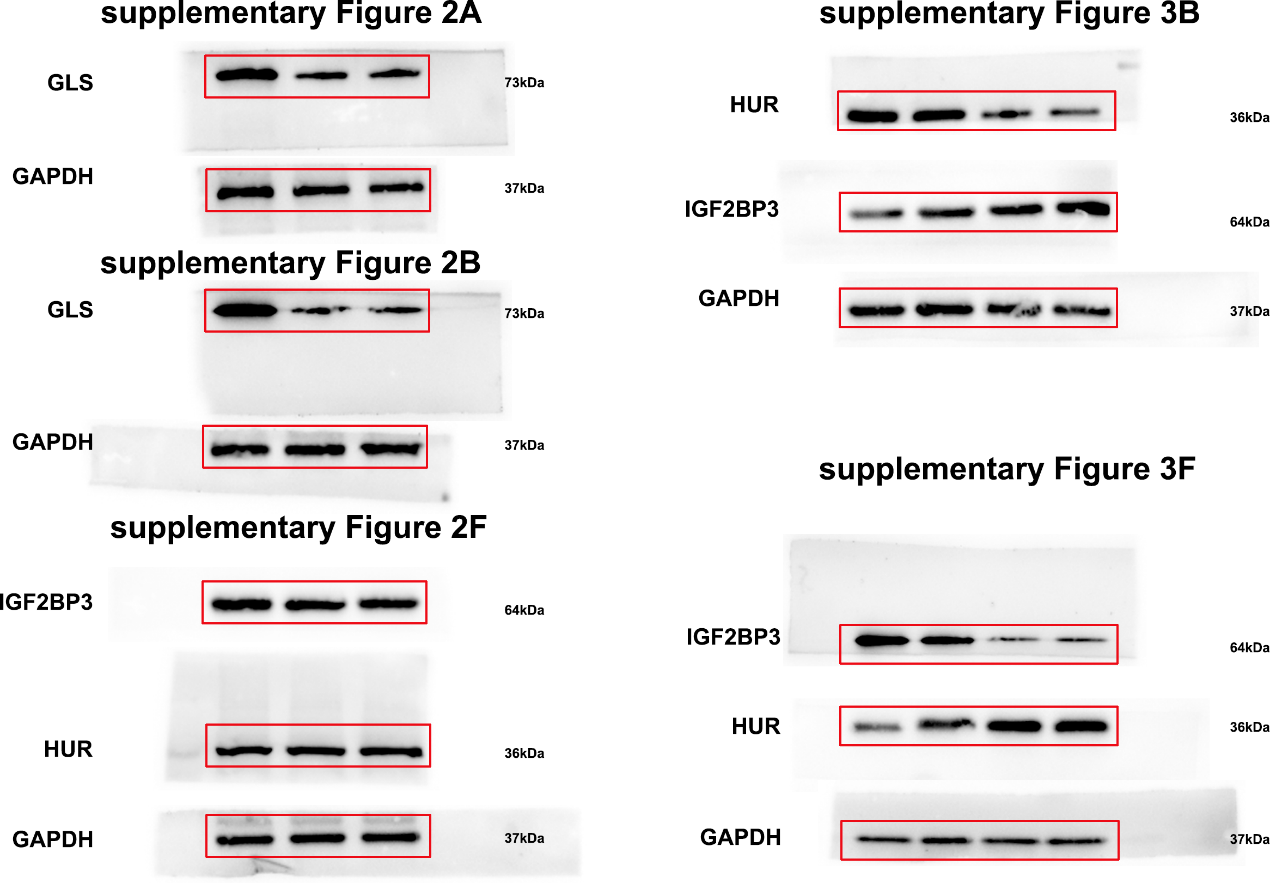


The uncropped scans of western blots and gels from the figures of the main manuscript.
