# Supplementary material for: m5C-methylated lncRNA NR_033928 promotes gastric cancer proliferation by stabilizing GLS mRNA to promote glutamine metabolism reprogramming
Source: Cell Death Dis. 2023 Aug 15;14(8):520. doi: 10.1038/s41419-023-06049-8 (PMC10427642; doi:10.1038/s41419-023-06049-8)
Supplement: Supplementary file 2 — supplementary materials [file 41419_2023_6049_MOESM2_ESM.docx]

**Supplementary Materials**

**Supplementary Figure S1**


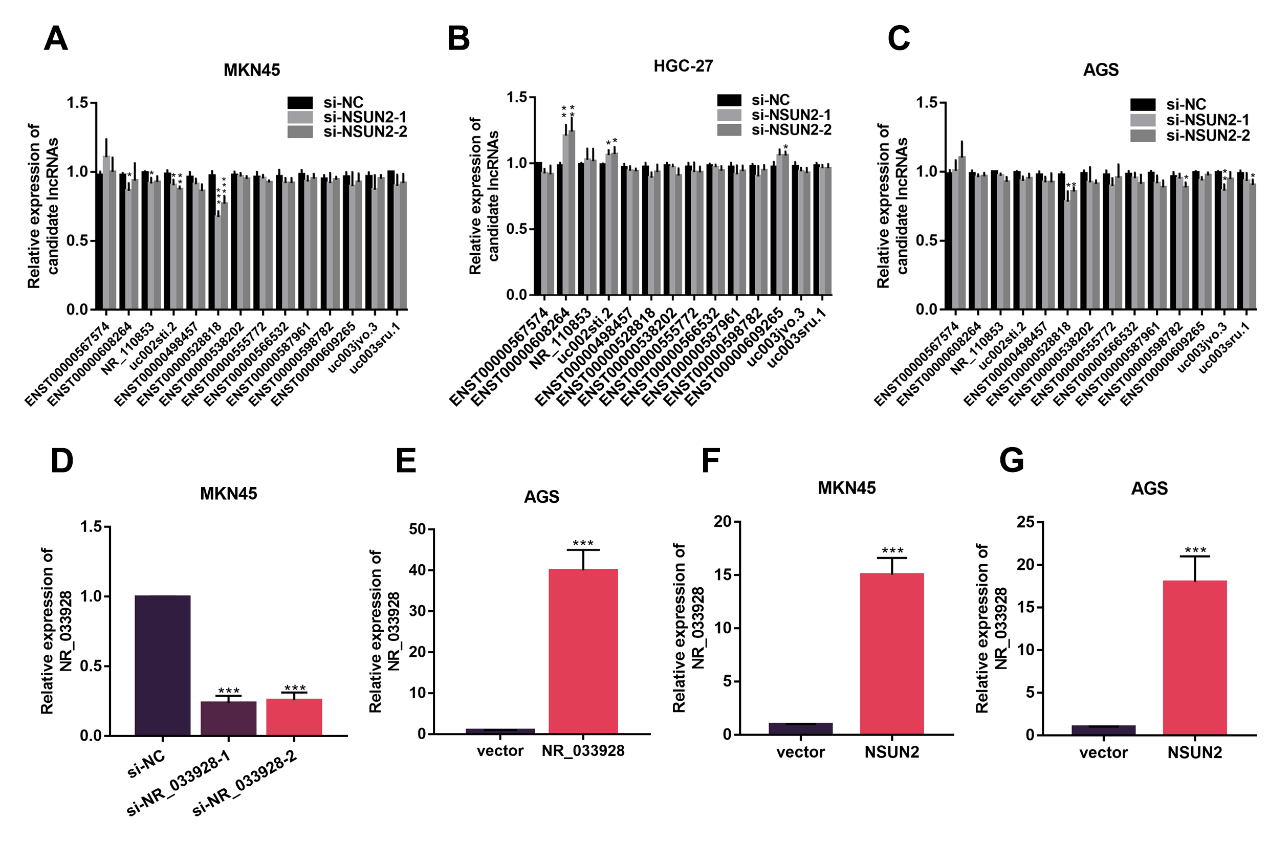


(A,B,C) qRT-PCR analysis of candidate lncRNAs expression in GC cells transfected with NSUN2 siRNAs.

(D,E) qRT-PCR analysis of NR_033928 expression in GC cells transfected with NR_033928 siRNAs and overexpressing vectors.

(F,G) qRT-PCR analysis of NR_033928 level in GC cells transfected with overexpressing NSUN2 vectors.

(Graph represents mean ± SD; *p< .05, **p< .01 and ***p< .001)

**Supplementary Figure S2**


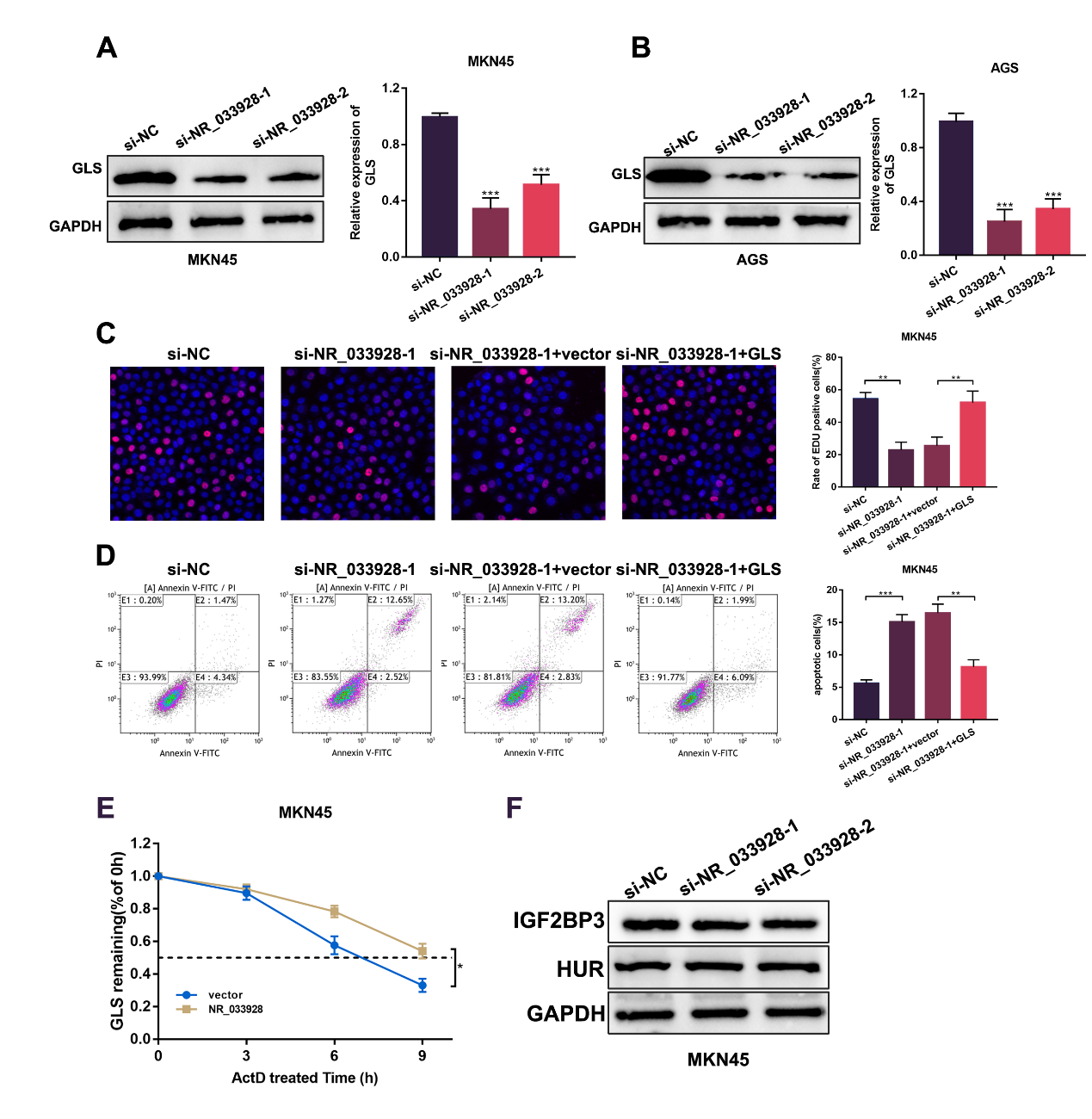


1. qRT-PCR analysis of relative GLS expression in MKN45 cells transfected with si-lnc.
2. Western blot analysis of GLS expression in MKN45 cells transfected with si-lnc.
3. EDU assays in MKN45 cells transfected with NR_033928 overexpressing vectors or co-transfected with sh-GLS lentivirus.
4. Apoptotic assays in MKN45 cells transfected with NR_033928 overexpressing vectors or co-treated with sh-GLS lentivirus.
5. RNA stability analysis of GLS level in cells transfected with NR_033928 overexpression vectors after treatment with 5 µg/mL actinomycin D.
6. Western blot analysis of the expression of IGF2BP3 and HUR in cells transfected with si-NR_033928.

(Graph represents mean ± SD; *p< .05, **p< .01 and ***p< .001)

**Supplementary Figure S3**


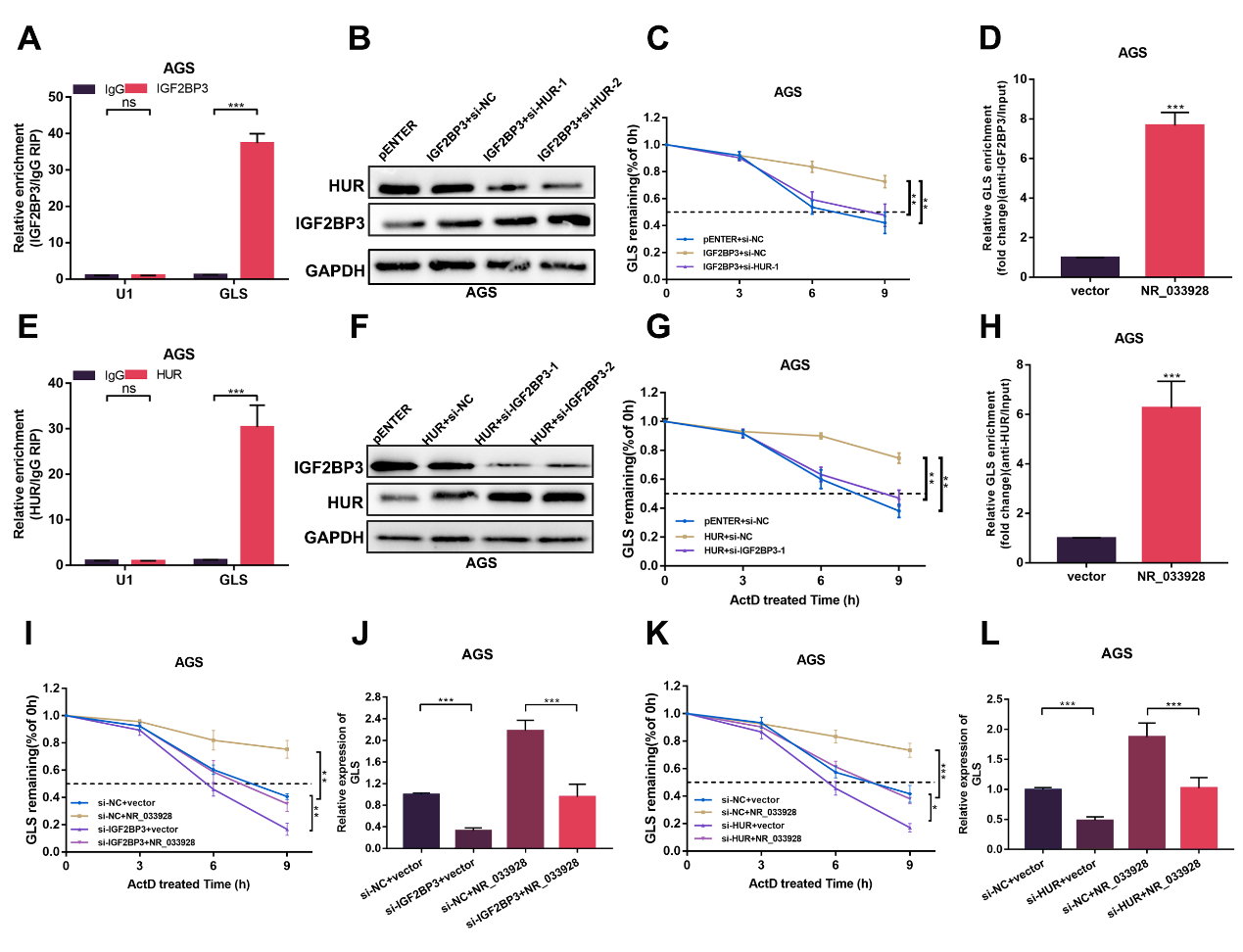


(A,E) RIP assays of relative GLS levels bound by IGF2BP3 antibody or HUR antibody.

(B) Western blot analysis of transfection of IGF2BP3 overexpression vectors and si-HUR in AGS cells.

(C) RNA stability analysis of GLS level in AGS cells transfected with IGF2BP3 overexpression vectors and si-HUR after treatment with 5 µg/mL actinomycin D.

(D, H) RIP assay of GLS bound by IGF2BP3 or HUR antibody in AGS cells transfected with NR_033928 siRNAs. qRT-PCR was used to detect the GLS expression.

(F) Western blot analysis of transfection of HUR overexpression vectors and si-IGF2BP3.

(G) RNA stability analysis of GLS level in AGS cells transfected with HUR overexpression vectors and si-IGF2BP3 after treatment with 5 µg/mL actinomycin D.

(I) RNA stability analysis of GLS level in AGS cells transfected with si-IGF2BP3 or co-transfected with NR_033928 overexpression vectors after treatment with 5 µg/mL actinomycin D.

(J) qRT-PCR analysis of GLS expression in AGS cells transfected with si-IGF2BP3 or co-transfected with NR_033928 overexpression vectors.

(K) RNA stability analysis of GLS level in AGS cells transfected with si-HUR or co-transfected with NR_033928 overexpression vectors after treatment with 5 µg/mL actinomycin D.

(L) qRT-PCR analysis of GLS expression in AGS cells transfected with si-HUR or co-transfected with NR_033928 overexpression vectors.

(Graph represents mean ± SD; *p< .05, **p< .01 and ***p< .001)

**Supplementary Figure S4**


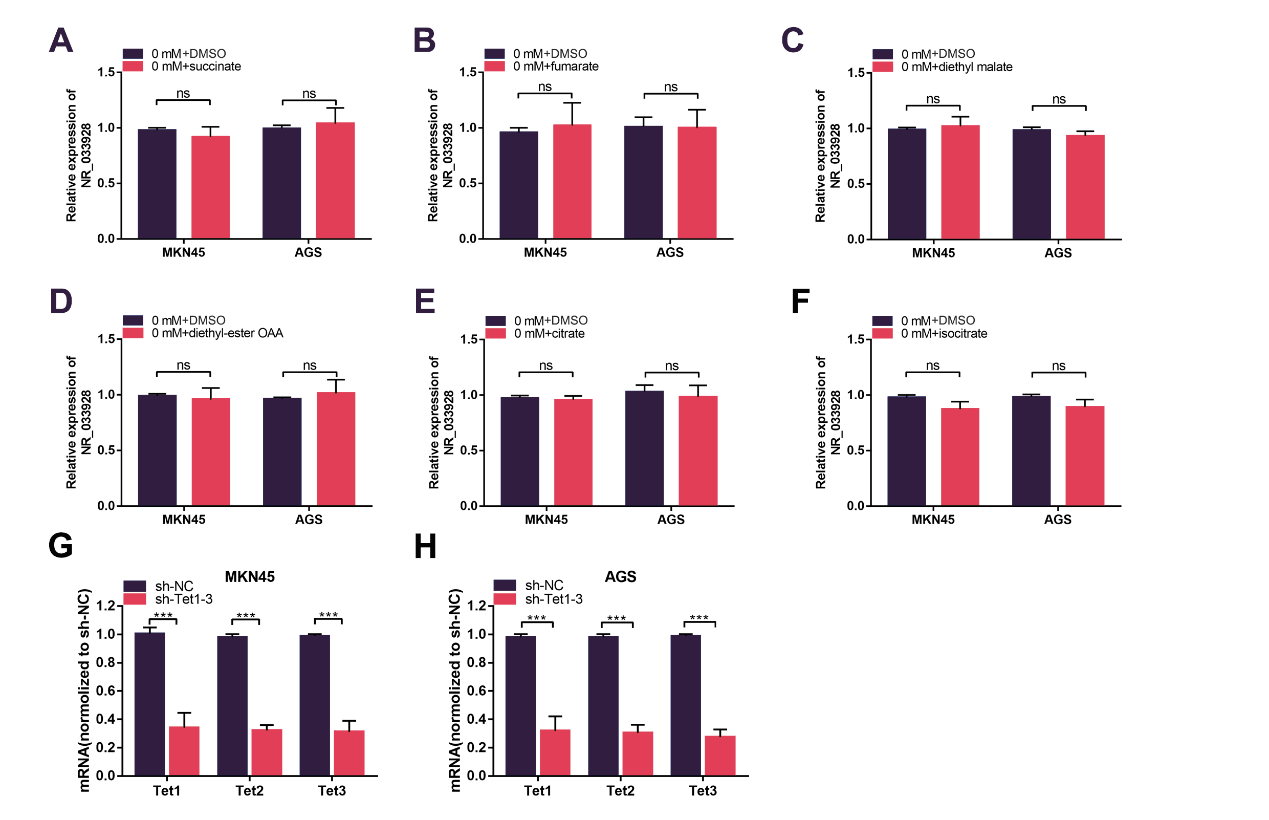


(A-F) qRT-PCR analysis of NR_033928 expression in GC cells in glutamine depletion medium after treatment of succinate (1mM 24H), fumarate (5mM 24H), diethyl malate (4mM 24H), diethyl-ester OAA (1mM 24H), citrate (4mM 24H), and isocitrate (1mM 24H).

(G,H) qRT-PCR analysis of the expression of Tet1, Tet2 and Tet3 in GC cells upon silencing of Tets family.

(Graph represents mean ± SD; *p< .05, **p< .01 and ***p< .001)

**Supplementary Figure S5**


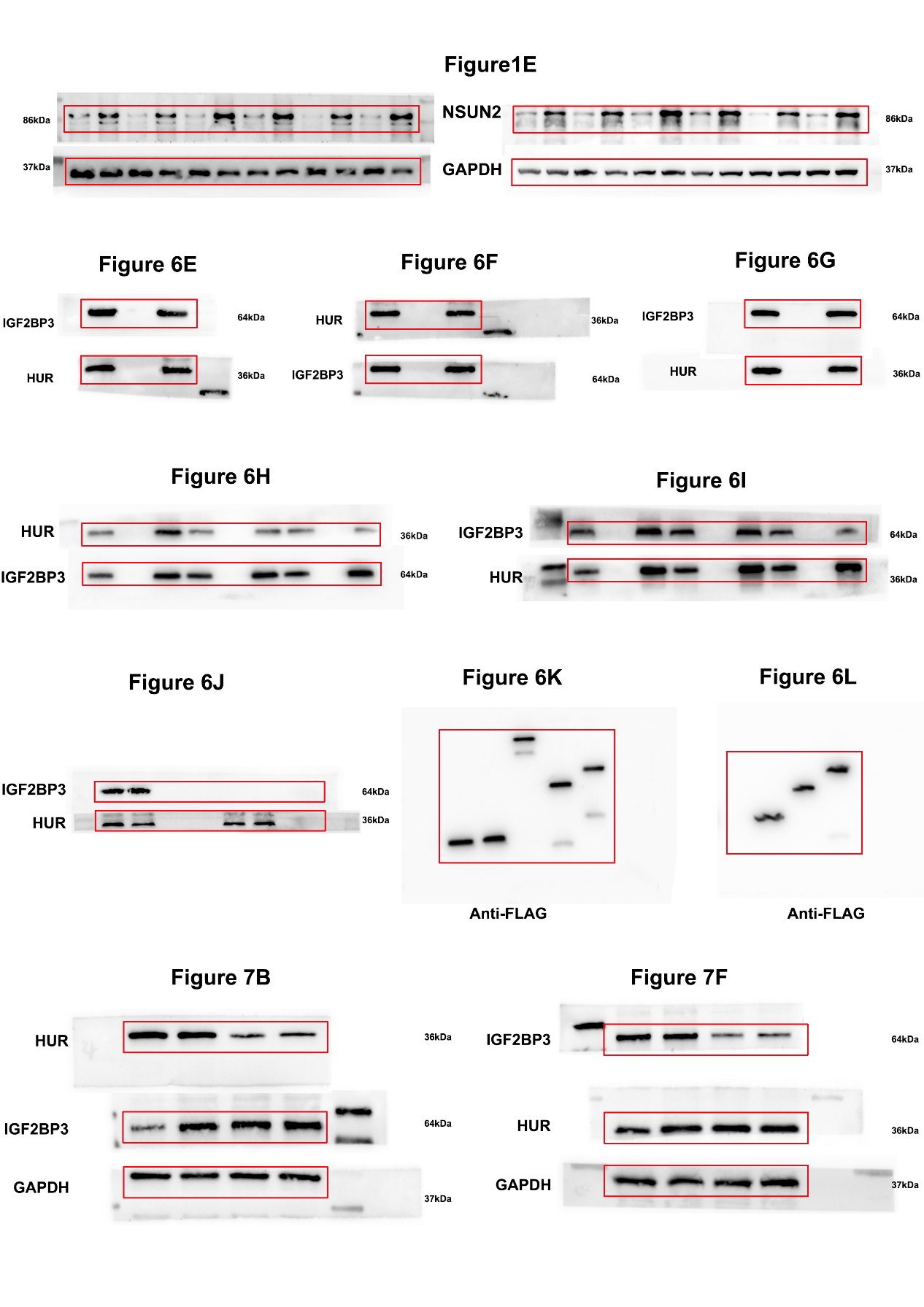


The uncropped scans of western blots and gels from the figures of the main manuscript.

**Supplementary Figure S6**


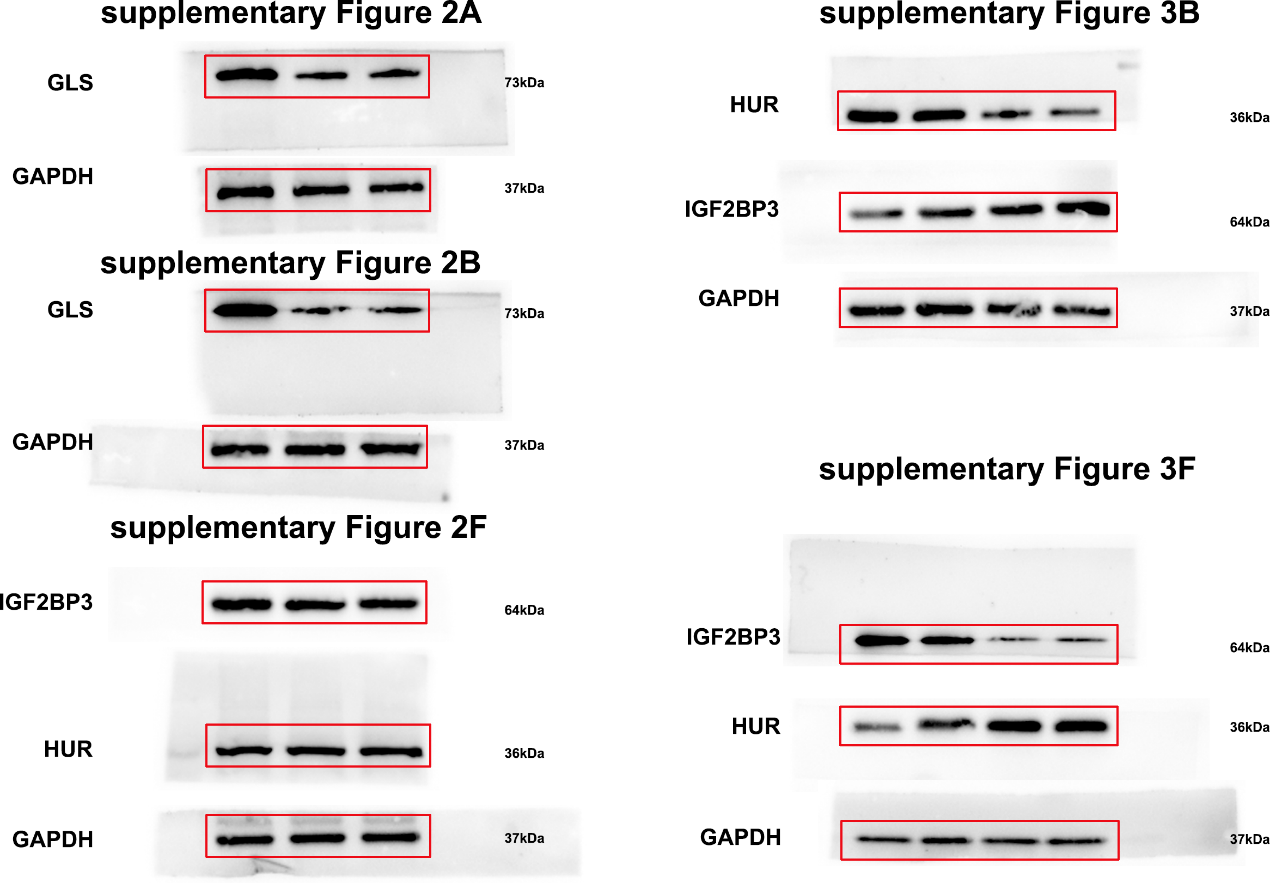


The uncropped scans of western blots and gels from the figures of the main manuscript.

|  |  | NR_033928 sense | | | | | | NR_033928 anti-sense | | | | | |
| --- | --- | --- | --- | --- | --- | --- | --- | --- | --- | --- | --- | --- | --- |
| UniProtKB  Accession | Gene Symbol | Unique Peptides #1 | Unique Peptides #2 | Unique Peptides #3 | Peptides #1 | Peptides #2 | Peptides #3 | Unique Peptides #1 | Unique Peptides #2 | Unique Peptides #3 | Peptides #1 | Peptides #2 | Peptides #3 |
| P07355 | ANXA2 | 35 | 34 | 35 | 35 | 34 | 35 | NA | NA | NA | NA | NA | NA |
| O00425 | IGF2BP3 | 31 | 34 | 31 | 33 | 37 | 36 | NA | NA | NA | NA | NA | NA |
| P11021 | HSPA5 | 34 | 33 | 27 | 37 | 36 | 30 | NA | NA | NA | NA | NA | NA |
| P08238 | HSP90AB1 | 29 | 29 | 26 | 49 | 46 | 40 | NA | NA | NA | NA | NA | NA |
| P10809 | HSPD1 | 39 | 32 | 26 | 39 | 32 | 26 | NA | NA | NA | NA | NA | NA |
| P0DMV9 | HSPA1B | 26 | 30 | 26 | 38 | 35 | 32 | NA | NA | NA | NA | NA | NA |
| P04083 | ANXA1 | 23 | 23 | 25 | 23 | 23 | 25 | NA | NA | NA | NA | NA | NA |
| P00558 | PGK1 | 32 | 23 | 25 | 32 | 23 | 25 | NA | NA | NA | NA | NA | NA |
| P04406 | GAPDH | 25 | 22 | 24 | 25 | 22 | 24 | NA | NA | NA | NA | NA | NA |
| Q15717 | ELAVL1 | 25 | 25 | 24 | 25 | 25 | 24 | NA | NA | NA | NA | NA | NA |

**Table S1. proteins identification results for NR_033928 sense in pull-down experiments by mass spectrometry**

**Table S2 Primers**

| NR033928 | Forward: 5’-ACGGGAGACTATCTGGCACATGA-3’  Reverse: 5’-TGGGAGTTCTTCGCAGGCAGGA-3’ |
| --- | --- |
| ENST00000498457 | Forward: 5’-AGCAACCTTCTGCCAGGATTT-3’  Reverse: 5’-CTTATTCCTCTGGAGTTTGAGGAGT-3’ |
| ENST00000528818 | Forward: 5’-CCATAGGGGCCACACACAAT-3’  Reverse: 5’-AGACAGGTGGATATATGTGGCAT-3’ |
| ENST00000538202 | Forward: 5’-GCTTACTGGTGCTGAGCAGA-3’  Reverse: 5’-GGAAGGAGATGCACACAAAGC-3’ |
| ENST00000555772 | Forward: 5’-GGGGTCTGAATGTTTTGCGG-3’  Reverse: 5’-GCGCCACCTCTAGTGTAGAA-3’ |
| ENST00000566532 | Forward: 5’-CCAGGAAGCAAGCTCAGTCT-3’  Reverse: 5’-AGAAACGGAAGACCCAGACA-3’ |
| ENST00000567574 | Forward: 5’-GGGATGCTGGTTGGTCTGTC-3’  Reverse: 5’-TTGTAACCGCTCTCACCAGG-3’ |
| ENST00000598782 | Forward: 5’-CAGTGGGGGCTACGATTGG-3’  Reverse: 5’-CAGCTAATGACACCCCGCAG-3’ |
| ENST00000587961 | Forward: 5’-TGGAAACGTGGAGTGGATGA-3’  Reverse: 5’-TCTTGCTTCCCAGGTCTCAG-3’ |
| ENST00000608264 | Forward: 5’-CAGGTAGGAGAAAAGAGGGGC-3’  Reverse: 5’-CTGACTGGTCTGGGTTAGGC-3’ |
| ENST00000609265 | Forward: 5’-GGGCCTTAGTCAGTTTGCCC-3’  Reverse: 5’-TCGAAGTGCCTGATTTGGGT-3’ |
| NR_110853 | Forward: 5’-GATGGGTCCGCTCTTTTCCA-3’  Reverse: 5’-GGAAGGGAGAGTTTGACCCG-3’ |
| uc002sti.2 | Forward: 5’-GATTGAGTGGCTTTGGTGGC-3’  Reverse: 5’-TCAGCGCCCATTACGTTTCT-3’ |
| uc003jvo.3 | Forward: 5’-GCATACCCTTTGTTTGGCGG-3’  Reverse: 5’-CTGGAAGCGTGTGGATGTCT-3’ |
| uc003sru.1 | Forward: 5’-TCCAGCCATGACTAAAGGGG-3’  Reverse: 5’-TTCCCTTTTGCATTGCCCTC-3’ |
| NSUN2 | Forward: 5’-GAACTTGCCTGGCACACAAAT-3’  Reverse: 5’-TGCTAACAGCTTCTTGACGACTA-3’ |
| GAPDH | Forward: 5’-TGTACCATCAATAAAGTACCCTGTG-3’  Reverse: 5’-AAATCCGTTGACTCCGACCT-3’ |
| GLS | Forward: 5’-TCCAGAAGGCACAG-3’  Reverse: 5’-AGACCAGCACATCATACC-3’ |
| Tet1 | Forward: 5’- CCTAGGACAGGCCTTTGGTG-3’  Reverse: 5’- CTGGGACAACACTCCCACTC-3’ |
| Tet2 | Forward: 5’- AGAGAATCCACCTGCAAGCT-3’  Reverse: 5’- TGGGGTGTGGCTATCAAGTT-3’ |
| Tet3 | Forward: 5’- CAACGGCTGCAAGTATGCTC-3’  Reverse: 5’- CTCGTTGGTCACCTGGTTCT-3’ |
| NR_0339928 promoter | Forward: 5’- GAAGAGCCCCACACTTTGCT-3’  Reverse: 5’- ACTCCGAATGCGAAGTTCTGT-3’ |
| si-NR033928-1 | Forward: 5’-GGUAAUCAGCCUCCCGAAATT-3’  Reverse: 5’-UUUCGGGAGGCUGAUUACCTT-3’ |
| si-NR033928-2 | Forward: 5’-GCCAGUGAAAUGUCACCUCTT-3’  Reverse: 5’-GAGGUGACAUUUCACUGGCTT-3’ |
| sh-NR033928-1 | 5’-GATCCGGTAATCAGCCTCCCGAAAT-3’ |
| sh-NR033928-2 | 5’-CAAGAGTTTCGGGAGGCTGAT-3’ |
| si-NSUN2-1 | 5’- GAAGCATCGTGCTGAAGTA -3’ |
| si-NSUN2-2 | 5’- GGGTTATCCTCACAAATGA -3’ |
| sh-GLS | 5’- GCACAGACATGGTTGGTATAT -3’ |
| sh-Tet1 | 5′- TTGTGCCTCTGGAGGTTATAA-3′ |
| sh-Tet2 | 5′- CAGATGCACAGGCCAATTAAG-3′ |
| sh-Tet3 | 5′-GCGATTGCGTCGAACAAATAG-3′ |

**Table S3 Antibodies and Recombinant proteins**

| GLS | abcam | ab260047 | WB/IHC |
| --- | --- | --- | --- |
| c-caspase3 | CST | #9661 | IHC |
| ki-67 | CST | #9449 | IHC |
| NSUN2 | Proteintech | 20854-1-AP | WB |
| IGF2BP3 | Proteintech | 14642-1-AP | WB |
| HUR | Proteintech | 11910-1-AP | WB |
| GAPDH | Proteintech | 60004-1-Ig | WB |
| GST-tagged HUR | Proteintech | Ag2249 | EMSA |
| HIS-tagged IGF2BP3 | ORIGENE | TP760798 | EMSA |

**Table S4 Probe Sequence for RNA pull-down and FISH**

| **RNA pull-down (5’-3’)** | ccgtggttgaggaacagaag |
| --- | --- |
| **FISH (5’-3’)** | tgggaccaagtggcaaaacg |
